# Unilateral optogenetic kindling of hippocampus leads to more severe impairments of the inhibitory signaling in the contralateral hippocampus

**DOI:** 10.3389/fnmol.2023.1268311

**Published:** 2023-10-24

**Authors:** Fabio Cesar Tescarollo, Daniel Valdivia, Spencer Chen, Hai Sun

**Affiliations:** Department of Neurosurgery, Rutgers Robert Wood Johnson Medical School, New Brunswick, NJ, United States

**Keywords:** optogenetics, kindling, inhibitory system, hippocampus, seizure

## Abstract

The kindling model has been used extensively by researchers to study the neurobiology of temporal lobe epilepsy (TLE) due to its capacity to induce intensification of seizures by the progressive recruitment of additional neuronal clusters into epileptogenic networks. We applied repetitive focal optogenetic activation of putative excitatory neurons in the dorsal CA1 area of the hippocampus of mice to investigate the role of inhibitory signaling during this process. This experimental protocol resulted in a kindling phenotype that was maintained for 2 weeks after the animals were fully kindled. As a result of the different phases of optogenetic kindling (OpK), key inhibitory signaling elements, such as KCC2 and NKCC1, exhibited distinct temporal and spatial dynamics of regulation. These alterations in protein expression were related to the distinct pattern of ictal activity propagation through the different hippocampal sublayers. Our results suggest the KCC2 disruption in the contralateral hippocampus of fully kindled animals progressively facilitated the creation of pathological pathways for seizure propagation through the hippocampal network. Upon completion of kindling, we observed animals that were restimulated after a rest period of 14-day showed, besides a persistent KCC2 downregulation, an NKCC1 upregulation in the bilateral dentate gyrus and hippocampus-wide loss of parvalbumin-positive interneurons. These alterations observed in the chronic phase of OpK suggest that the hippocampus of rekindled animals continued to undergo self-modifications during the rest period. The changes resulting from this period suggest the possibility of the development of a mirror focus on the hippocampus contralateral to the site of optical stimulations. Our results offer perspectives for preventing the recruitment and conversion of healthy neuronal networks into epileptogenic ones among patients with epilepsy.

## Introduction

Temporal lobe epilepsy (TLE) is the most common type of epilepsy affecting adults worldwide (Tellez-Zenteno and Hernandez-Ronquillo, 2012; Fiest et al., 2017). Approximately 20–40% of diagnosed TLE cases are highly refractory to anti-seizure drugs (ASDs) and are associated with an increased risk of morbidity and mortality among patients with medically intractable seizures (Engel, 2014). TLE seizures originate from small neuronal clusters in the hippocampal formation and can become secondarily generalized. Seizure generalization occurs through ictal activity propagation through healthy cerebral tissue, ultimately affecting both cerebral hemispheres (Blumenfeld et al., 2009). The recurrence of seizure generation and generalization cycle leads to the recruitment of initially uninvolved neural populations in the epileptogenic network, resulting in a gradual increase in the seizure severity and duration -- a phenomenon known as kindling (Luders, 2001; Kim et al., 2014; Liu et al., 2020). The development of TLE is attributed to an abnormal decline in inhibitory signaling and a concomitant increase in excitatory neurotransmission within the affected neural substrates, particularly in the hippocampus (Sloviter, 1987; de Lanerolle et al., 1989; Kamphuis et al., 1991; Lau et al., 2000; Cossart et al., 2001; Bonansco and Fuenzalida, 2016; Righes Marafiga et al., 2021). Similarly, the kindling process is a result of the progressive weakening of the inhibitory system (Kamphuis et al., 1991; Bertram, 2007; Ryu et al., 2021). Since the role of a dysfunctional inhibitory system in the recruitment of additional neuronal clusters during the kindling process is still poorly understood, we have implemented a murine OpK model to study this process.

Electrical kindling (EK) is one of the most used models for preclinical evaluations of ASDs (Löscher, 2002) and has been extensively employed by researchers to study TLE (McNamara, 1986). Recent studies have established OpK as a promising alternative to the classical EK. Advantages of an OpK model include the ability to selectively target specific neuronal subpopulations and the lack of artifacts from electrical stimulation during the local field potential (LFP) recordings (Cela and Sjostrom, 2019; Butler et al., 2020; Choy et al., 2021; Ryu et al., 2021; Shimoda et al., 2022).

We selectively activated glutamatergic neurons in the unilateral hippocampal *Cornus Ammonus*-1 (CA1) area of mice using optogenetics to evaluate changes in key components of inhibitory signaling in response to kindling. Specifically, we investigated the inhibitory components that are involved in regulating GABAergic transmission. One key element of GABAergic transmission is the intracellular chloride ([Cl^−^]i) homeostasis (Achilles et al., 2007; Liu et al., 2019), which is maintained by two main cation-chloride cotransporters (CCCs), i.e., the neuron-specific outwardly directed K^+^–Cl^−^ cotransporter 2 (KCC2) (Rivera et al., 1999) and the inwardly directed Na^+^, K^+^–2Cl^−^ cotransporter 1 (NKCC1) (Otsu et al., 2020). We also assessed whether the OpK led to parvalbumin-positive interneuron (PV-INs) loss. Pathological alterations in KCC2 and NKCC1 expression accompanied by the loss of PV-INs have been identified as markers in epilepsy patients (Sen et al., 2007; Kahle et al., 2016; Liu et al., 2019). Here, we reported the temporal and spatial alterations of NKCC1, KCC2, and PV-INs as a result of kindling in specific sublayers of the bilateral hippocampus. Furthermore, we combined multichannel LFP recordings with computational techniques to evaluate the hippocampal network responses during the progression of kindling and showed the patterns of ictal activity in each hippocampal sublayer reflect the degree of impairment of inhibitory signaling observed.

## Materials and methods

### Animals

Experiments were carried out with adult (8–10 weeks old) male and female inbred homozygous PV-Cre mice (B6.129P2-Pvalb<tm1(cre)Arbr>/J, Strain #: 017320 – The Jackson Laboratory). The mice were reared under social housing and environmental enrichment conditions, with food and water provided *ad libitum* under standardized temperature, humidity, and a 12 h light/dark cycle (lights on from 6:00 am to 6:00 pm). All animal studies were conducted in accordance with approved Rutgers Institutional Animal Care and Use Committee (IACUC) protocols within an Association for Assessment and Accreditation of Laboratory Animal Care (AAALAC) accredited facility.

### Viral vector injection and optrode implantation

Animals were stereotaxically injected with 300 nL of pAAV-5-CamKIIα-hChR2(H134R)-eYFP (ChR2) in the hippocampal CA1 layer unilaterally at a rate of 30 nL/min (Addgene viral prep # 26969-AAV5; RRID: Addgene_26969, titer: 2.2×10^13^ genome copies/ml). The injection was performed with a Hamilton syringe (Neuros 7001, Hamilton Co., Reno, NV, USA) and an automatized injector (Quintessential Stereotaxic Injector, Stoelting Co., Wood Dale, IL, USA). Injection coordinates for the dorsal hippocampal CA1 from bregma were: anteroposterior (AP) = −2.06 mm, mediolateral (ML) = −1.6 mm, and dorsoventral (DV) = −1.37 mm. After the injection of the ChR2 viral vector, the needle was maintained in place for 5 min to avoid backflow and then slowly retracted.

**Figure 1:**
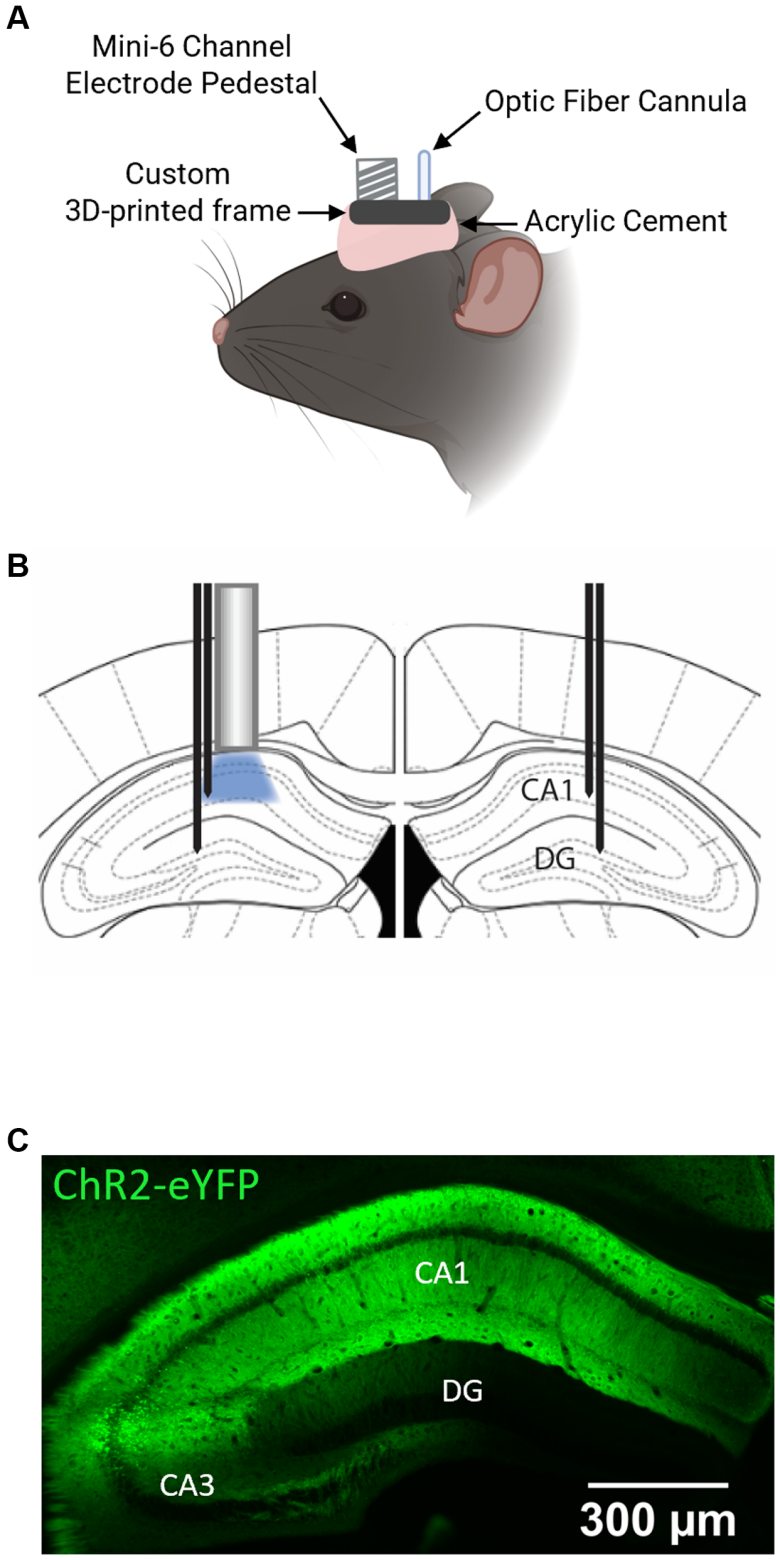
ChR2-eYFP expression and optrode placement in the mouse hippocampus. **(A)** Schematic illustration of the head-stage ensemble in a mouse. Created with Biorender.com. **(B)** Schematic of the placement of optrode above left CA1 and recording electrodes in bilateral CA1 and DG. **(C)** Distribution of ChR2-eYFP in the hippocampus of PV-Cre^+/+^ mouse. Note the high expression levels in the CA1.

In the same surgery, animals were implanted with an optrode ensembled in a custom 3D-printed frame (see Figure 1A), composed of an optic fiber cannula (0.66 NA, 400 mm diameter) for laser stimulations (Doric Lenses, Quebec – Canada) and a 6-channel pedestal (Mini6, Plastics One, Roanoke, VA, USA), that was interfaced between the electrodes and the recording system. Four recording electrodes (F12146, P1 Technologies, Roanoke, VA, USA) were positioned at bilateral CA1 (AP, −2.06 mm; ML, ±1.6 mm; DV, −1.4 mm) and DG (AP, −2.06 mm; ML, ±1.6 mm; DV, −1.9 mm) (see Figure 1B for optic fiber and recording electrodes arrangement in the bilateral hippocampus). The tip of the fiber cannula was implanted just above the viral vector injection site (AP, −2.06 mm; ML, −1.6 mm; DV, −1.1 mm). The two remaining electrodes implanted were connected as ground and reference. The ensemble was secured to the skull using dental acrylic cement. Anesthesia was induced with isoflurane (~2.5%). The animals were transferred to a heating pad and kept warm while anesthesia was maintained throughout the whole surgical procedure (~1.5% during maintenance). Bupivacaine (2.5 mg/kg, s.c.) was administered at the incision site for local anesthesia. Sustained release buprenorphine (EthiqaXR, 3.25 mg/kg, s.c.) was administered to alleviate pain and discomfort. After surgery, the animals were individually housed in a clean cage to recover. Experiments were performed at least 21 days after the surgical procedure to allow time for viral-induced ChR2 expression. A typical pattern of ChR2 expression in the hippocampus of the animals used in the experiments is shown in Figure 1C.

For control, a separate group of animals was injected with a blank ChR2 virus (pAAV-5-CamKIIα-eYFP, titer: 1.9×10^13^ genome copies/ml) and underwent the same surgical procedures.

### OpK protocol and experimental cohorts

**Figure 2:**
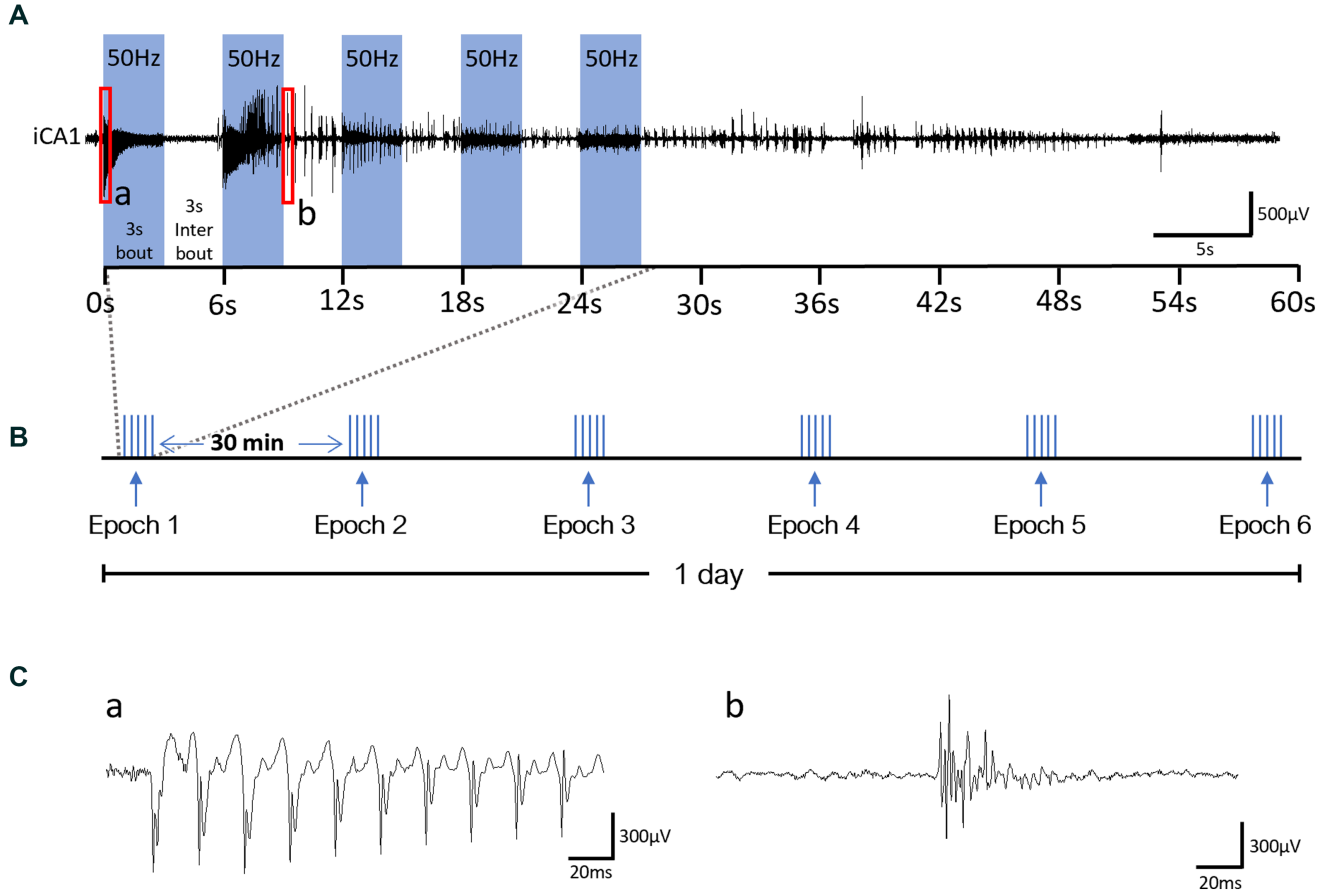
Optogenetic Kindling stimulation protocol schematic and oADs from a representative Day 1 recording. **(A)** Example of an LFP recording from the CA1 layer of the hippocampus ipsilateral to the optical stimulation and schematic of one epoch of stimulation. 50 Hz laser stimulation was delivered unilaterally to CA1 in 3 s-bouts (blue bars). Bouts were delivered 5x and interleaved by 3 s of no-stimulation (3 s-interbout). **(B)** Schematic of our OpK protocol for 1 day of stimulation. Six epochs were delivered every 30 min to the animals. **(C)** Magnified segments of electrographic responses evoked by the laser stimulation from a and b labeled red rectangles in panel **(A)**; insert a: optogenetically-evoked population discharge (oPD) and b: optogenetically-evoked after-discharge (oAD).

Our OpK protocol consisted of daily laser stimulation epochs until each animal became fully kindled, which was defined by the seizure severity score observed in response to the stimulation epochs. Each epoch consisted of five 3-s stimulation bouts, with each bout consisting of laser pulsing delivered at 50 Hz with 25% duty cycle and 1.2 mW max power, separated by a 3-s rest inter-bout (Figure 2A). The stimulation epochs were delivered six times per day, separated by a 30-min rest period between epochs (Figure 2B). In the case of an animal displayed 3 consecutive Racine Score 7 seizures, the stimulations were halted. We used a modified Racine scale (RS) adapted from Van Erum et al. (2019), to score the behavioral severity of seizures from the videos: RS0 – no behavioral alteration, RS1 – arrest or sudden motion, RS2 – mouth chewing and/or head nodding, RS3 – forelimb clonus, RS4 – fore- and hindlimb clonus + rearing, RS5 – loss of posture/falling, RS6 – brief wild running or jumps; RS7 – severe wild running or jumps. The experimental cohorts were established based on the premise that the development of kindling entails a progressive intensification of seizure severity until a maximum seizure severity level (plateau) is reached and maintained (Bertram, 2007). The first experimental cohort, referred to as the kindled (Ki) group, was comprised of fully kindled animals, or animals that exhibited three seizures with maximum severity level (RS7) in response to three consecutive stimulation epochs in the kindling phase. The second cohort, referred to as the rekindled (Rek) group, was comprised of animals that were fully kindled, maintained without any stimulation for 14 days, and then restimulated until the animals exhibited three RS7 seizures in response to three consecutive stimulation epochs. The rekindling protocol was identical to the kindling protocol. Two additional cohorts of animals were used as control for optogenetics. The animals in these cohorts underwent the same OpK protocol during the average number of stimulation epochs that animals from the Ki experimental group underwent (30 epochs). In the first control group (Sham), the animals were injected with a ChR2 viral vector but were stimulated with mismatched laser stimulations (wavelength: 590 nm, 1.2 mW). Animals from the Sham group were used as control animals for the histologic evaluation of KCC2 and NKCC1 expression, and PV-INs loss. In the second control group, the animals were injected with a blank viral vector and stimulated with the blue laser (wavelength: 470 nm, 1.2 mW).

Based on prior studies, a series of preliminary experiments were conducted to determine the stimulation parameters that were most effective in consistently evoking oADs in response to subthreshold laser stimulation (data not shown). To determine the stimulation mode and frequency a cohort of animals (*n* = 4) was submitted to laser stimulations every 30 min using the following parameters: (i) continuous 20 Hz laser stimulation for 15 s, adapted from Lothman and Williamson (1993), (ii) continuous 50 Hz laser stimulation for 15 s, (iii) interleaved (3 s-on/3 s-off), adapted from Cela et al. (2019), 20 Hz stimulation for 30 s, and (iv) interleaved (3 s-on/3 s-off) 50 Hz stimulation for 30 s. The final protocol parameters were chosen based on this limited assessment; the full parametric space is yet to be explored.

To determine the threshold power output of the laser, another cohort of animals (*n* = 4) underwent laser stimulation using the parameters used in the present study. Stimulation epochs were delivered every 30 min beginning from 0 mW of laser power. The power was gradually increased by 0.2 mW at a frequency of 50 Hz in each epoch until optogenetic-evoked after discharges (oADs) (Klorig et al., 2019) were observed. The average laser power required to elicit oADs in each animal was then calculated and approximated.

### Optogenetic stimulations and *in vivo* video-EEG recordings

For the LFP recordings, each animal was placed in the recording cage with a cable connected to the optrode/electroencephalogram (EEG) ensemble. Simultaneous optical stimulation and EEG recording were conducted. Optical stimulation was delivered by a 470 nm blue laser (LMS-BY02-GF3-00020-05 dual laser unit, LaserGlow Technologies, Toronto, ON, Canada). The light power exiting the tip of the optic patch cord, immediate to the animal connection, was measured using an optical power meter (PM-20A, Thorlabs Inc., Newton, NJ, USA) and set to 1.2 mW while delivering 5 ms pulses at 50 Hz and 25% duty cycle, before each day of optical stimulations. Taking into consideration that 1 mW/mm^2^ is required for ChR2 activation (Aravanis et al., 2007), we calibrated the light power level delivered by the optic fiber to allow a maximum brain tissue volume of ~0.1 mm^3^ directly activated by the laser stimulation for our optic fiber, calculated using an online tool.^1^ A 590 nm orange laser was configured similarly for sham stimulation among control animals.

EEG signals were collected at 6 kHz using a PZ5/RZ5 system (Tucker-Davis Technologies, Alachua, FL, USA). The same system also controlled the laser stimulations and video acquisition. EEG traces were reviewed in a custom Matlab application (MathWorks, Natick, MA, USA) and visually evaluated for optogenetically-evoked population discharges (oPDs), oADs, and seizure activity (Figure 2A, see insert b).

Baseline activity was recorded for 5 min before the first stimulation epoch and for another 5 min after the last seizure evoked in each stimulation day.

### Histology processing

For histological analysis, mice were deeply anesthetized with isoflurane and perfused transcardially with 120 mL of 4% paraformaldehyde (PFA) in 1X phosphate buffer (PBS – pH7.4) 24 h after the animals had reached three consecutive RS7 seizures after undergoing the kindling process (Ki), or three consecutive RS7 seizures after undergoing rekindling (Rek). Animals from the Sham group, stimulated with a mismatched laser wavelength, were perfused 24 h after undergoing the average number of epochs that animals from the Ki experimental group (30 epochs). Brains were extracted and post-fixed for 24 h in 4% PFA and then transferred to 30% sucrose with 0.02% sodium azide in 4% PFA for 2 days at 4°C. Coronal brain sections of 30 μm thickness were made on a cryostat (Leica CM3050S, Leica Microsystems, Wetzlar, Germany) and were stored in cryoprotectant at −20°C until further processing was initiated.

Coronal sections of the hippocampi were used to evaluate the ChR2 expression and to conduct immunofluorescence staining for KCC2 and NKCC1, and immunohistochemistry staining for PV. For ChR2 expression assessment, sections containing the dorsal hippocampus were mounted on glass slides, cover-slipped with DAPI mounting medium, and stored in the dark at 4°C until images were acquired. For each staining routine targeting PV, KCC2, and NKCC1 expression, we selected six sections from each animal with an approximately uniform caudo-rostral location to cover the whole anterior–posterior extent of the hippocampus. AP distance of the slices from Bregma were approximated at the following slice intervals: Slice 1: 1.30–1.65 mm, Slice 2: 1.65–2.00 mm, Slice 3: 2.00–2.35 mm, Slice 4: 2.35–2.70 mm, Slice 5: 2.70–3.10 mm, and Slice 6: 3.10–3.50 mm.

For PV immunohistochemistry, the coronal brain sections were processed as free-floating sections. Brain sections were washed 5x5min in a phosphate-saline buffer solution (PBS) and then incubated for 30 min in 0.3% H_2_O_2_. Sections were washed 3 × 10 min in PBS containing 0.05% Triton X-100 (PBST) at room temperature and blocked for 1 h using 10% Donkey Blocking Buffer (DBB). The sections were then incubated overnight at 4°C in DBB containing the primary antibody, polyclonal rabbit anti-PV (1,5000 –#PA1-933, Invitrogen, Waltham, MA, USA). Sections were washed 3x10min with PBST and then incubated in 10% DBB containing biotin-conjugated goat anti-rabbit IgG secondary antibody (1,5000 –#SAB4600006, Sigma-Aldrich, St. Louis, MO, USA). After washing 5×10 min in PBST, sections were incubated in 3,3’-Diaminobenzidine (DAB) substrate solution (SK-4105; Vector Laboratories, Newark, CA, USA) for up to 10 min until the reaction product was visualized. Sections were washed 3×5 min in PBS, mounted on slides, and then allowed to dry overnight. Sections were dehydrated in a series of alcohol baths, cleared in xylene, and cover-slipped with Permount-G mounting medium (SP15-100 UN1294; Fisher Scientific, Waltham, MA, USA). Bright-field images were acquired using a Leica light microscope (Leica DM 4B, Leica Microsystems, Wetzlar, Germany). Images were acquired using an image screening function at 10x magnification on a brightfield Leica microscope (Leica DM 4B, Leica Microsystems, Wetzlar, Germany) for PV-INs cell counting, allowing for higher cell resolution in the images. Cell counting was performed using the particle analyzer function of ImageJ (ImageJ, U.S. National Institutes of Health, Bethesda, MD, USA). For KCC2 or NKCC1 immunofluorescence, free-floating brain sections were washed 3x5min in tris-buffered saline (TBS) containing 0.05% of Triton X-100 (TBST) and blocked for 1 h at room temperature using DBB. The sections were incubated overnight at 4°C in DBB containing the primary antibodies rabbit anti-KCC2 (1,500, Millipore, #07432) or rabbit anti-NKCC1 (1,500, #13884-1-AP, Proteintech, Rosemont, IL, USA). Sections were washed 3×10min in TBST and incubated in DBB containing the fluorescent secondary antibodies Donkey anti-rabbit Alexa Fluor 633 (1,1000, A-21202, Thermo Fisher, Waltham, MA, USA). After 3 × 10 min washes in PBS, the processed sections were mounted on slides, cover-slipped with DAPI mounting medium, and then stored in the dark at 4°C. Fluorescent images were acquired on a Leica fluorescent microscope (Leica DM 4B; Leica Microsystems, Wetzlar – Germany) and presented single channel acquisitions. The protein levels of KCC2 and NKCC1 protein expression were quantified as relative fluorescent intensity (RFI) over a selected region of interest (ROI) using ImageJ software (ImageJ, U.S. National Institutes of Health, Bethesda, MD, USA). The region of interest (ROI) measured was: (a) the entire bilateral hippocampus (global expression), and (b) individual unilateral hippocampal sublayers (iCA1, cCA1, iCA3, cCA3, iDG, and cDG). The RFI for each slide was background corrected by subtracting the RFI of the background measured over an ROI selected from an empty area of the slide.

### EEG coastline index calculation

The coastline index (CI) was calculated to quantitatively assess the levels of EEG activity in each hippocampal recording channel in response to the progression of kindling. The CI algorithm employed in this study (shown below) measures the size of the EEG voltage change from sample to sample per unit of time (expressed in V/s, and was shown to be sensitive to quick and high fluctuations of ictal activities (Niknazar et al., 2013; Berglind et al., 2018).


$$\mathrm{Coastline}\left[t\right]=\;\left|eeg\left[t\right]-eeg\left[t-1\right]\;\right|$$

The coastline index for each electrode and epoch was baseline adjusted such that the average pre-stimulus coastline was set to 0 V/s (*t* = −6 s to *t* = 0 s, where *t* = 0 is the start of each epoch); subsequently, the negative adjusted coastline index was reset to 0 V/s. The EEG activity trend over each epoch was summarized in 6 s intervals – corresponding to a full 6 s laser stimulation cycle – by taking the average coastline index over each 6 s period from *t* = −6 s to *t* = 60s.

### Correlation analysis

An overall EEG aggravation index at each electrode was estimated by integrating the 6 s coastline indices across all seizures from the same animal and expressed as average V/s. We correlated this EEG aggravation index with the KCC2 and NKCC1 expression levels using the RFI obtained for each CCC analyzed. We examine this relationship at the network level by plotting the average EEG aggravation index of all animals against the MGI of each protein hippocampal sublayer (iCA1, cCA1, iDG, and cDG. At the individual (animal) level, we examined Pearson’s correlation coefficient *r* between the EEG aggravation index and the expression level of each protein (in RFI units) for each hippocampal sublayer (iCA1, cCA1, iDG, and cDG) and for each experiment cohort (Ki and Rek).

### Statistical analysis

Statistical analysis was conducted using GraphPad Prism 8 (Dotmatics, Boston, MA, USA). Values are reported as the mean ± SD unless stated otherwise. The statistical analysis for KCC2 and NKCC1 protein expression was performed on the raw RFI values. The RFI values obtained from each ROI were normalized to the respective ROI of the Sham group and expressed as a percentage of the increase or decrease of protein expression relative to Sham. The Wilcoxon matched-pairs signed-rank test was used to compare median differences. A one-way and two-way analysis of variance (ANOVA) was used for multiple comparisons. Tukey’s and Dunnett’s non-parametric tests were used for *post hoc*, pairwise multiple comparisons. The Pearson correlation was used to test the linear relationship between variables. A *p*-value smaller than 0.05 was considered significant.

## Results

### Analysis of the electrographic and behavioral changes in response to repetitive dorsal CA1 optical stimulation

#### EEG pattern and seizure severity on the first day of optical stimulation

**Figure 3:**
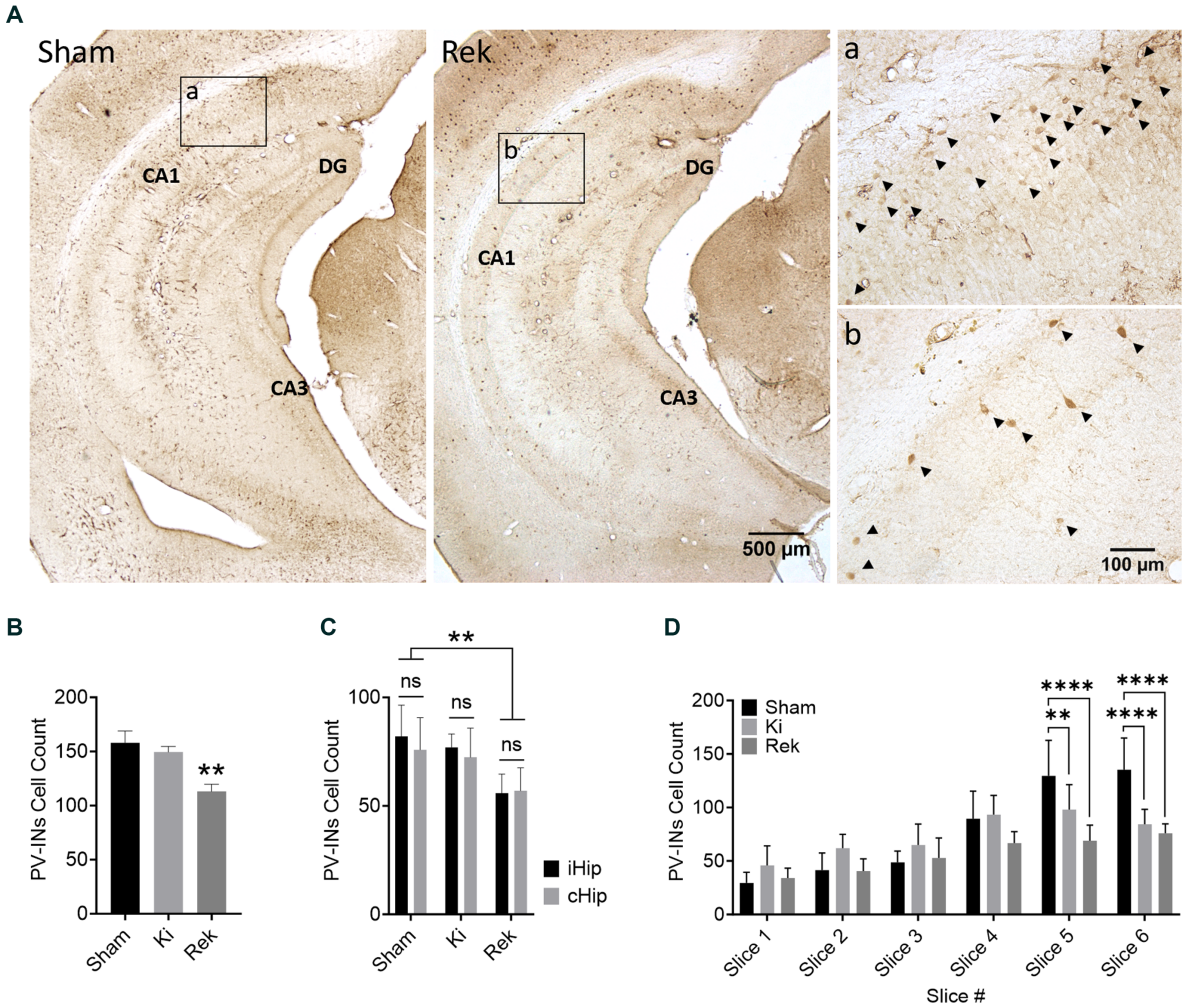
Establishment and retention of OpK. **(A)** Progression of OpK across multiple days of laser stimulation. Each dot in the graph represents the Mean ± SEM of the Racine score observed in each epoch across the animals (*n* = 22). Six stimulation epochs were delivered to the animals each day. Note that seizure severity intensified within each day of stimulation and across the kindling period. OpK was achieved and stimulation halted when each animal showed 3 consecutive Racine score 7 seizures within the same day, represented by the blue dots. The red dots represent the seizure severity from the first epoch of each day. After 14 days of idleness, the OpK protocol was resumed in eight fully kindled animals (the rekindling period). During the rekindling period, seizure severity showed a fast progression (black triangles) toward 3 consecutive Racine score 7 seizures (green triangles). **(B)** Correlation between the seizure duration and seizure severity across the OpK (Mean ± SEM, *r* = 0.9864, *p* < 0.0001, Pearson’s *r* correlation test). **(C)** Comparison of the number of epochs necessary to evoke 3 consecutive Racine score 7 seizures between the Kindling (Ki) and Rekindling (Rek) period (****p < 0.0001 in relation to Ki, paired Student’s *t*-test).

During the OpK experiments, the first stimulation epoch delivered to each mouse (*n* = 22) resulted in an immediate hippocampal activation time-locked with laser pulses. This is termed optogenetically-induced population discharge (Figure 2C, insert a; Klorig et al., 2019). At varying times within each epoch, oPDs were gradually interleaved by spontaneous activity, namely optogenetically-induced after discharges (oAD), until the oPDs were replaced by oADs (Figure 2C, insert b). All the animals included in this study showed oPDs and oADs in response to the optical stimulations within the first epoch of the first day. Behavioral seizures started emerging after an average of 3.5 ± 2.1 epochs across the mice, and seizure severity on the first day ranged from RS 0 to 3 (Figure 3A).

#### Increased seizure severity across days

All animals exhibited a progressive increase in the seizure severity (Figure 3A, kindling period), and duration (Figure 3B, *r* = 0.9864, *p* < 0.0001, Pearson’s r Correlation Test) as result of repeated stimulation. The stimulation epochs were delivered 6 times per day until the animals became fully kindled (Figure 3A; Bertram, 2007). The animals became fully kindled in an average of 23.5 ± 3.3 stimulation epochs (range: 17 to 30).

The evocation of after-discharges is required on every stimulation delivered for the kindling phenomenon to be produced experimentally in animals (Goddard et al., 1969; Racine, 1972). Here, every stimulation epoch delivered to the animals resulted in an oAD. Interestingly, the first epoch of each day conferred a significant reduction in the seizure severity in comparison with the last epoch of the previous day. The average reduction in the RS was 2.3 ± 2.9 points, ranging from 0 to 6 (Figure 3A, red dots). However, this reduction was confined only to the first epoch of the day, and it did not impede the progression of kindling. The seizures observed on the subsequent epoch of the same day promptly reached or even surpassed the seizure severity evoked in the last epoch of the previous day, showing a clear progression of the behavioral severity across the OpK protocol (Figure 3A).

#### Animals remained chronically kindled

We assessed whether the increased seizure susceptibility from OpK persisted in the long term after the stimulations had stopped. Accordingly, a subset of fully kindled mice was re-stimulated after 14 days free of stimulation: the Rek group (*n* = 8). The stimulation parameters employed during the rekindling period were the same as those applied during kindling. All eight animals displayed behavioral responses to stimulations similar to their last day of stimulation before rest (Figure 3A, rekindling period). The first epoch of rekindling resulted in low seizure severity similar to the pattern of responses during the kindling period. The average RS for the first epochs in the kindling period was 2.3 ± 1.2, whereas the average RS for the first epochs in the rekindling period was 1.8 ± 2.1 (*p* = 0.3750, Wilcoxon matched-pairs signed-rank test). We observed a much faster progression in the seizure severity during the rekindling period compared to the kindling period. It required an average of 23.5 ± 3.3 stimulation epochs (range: 17 to 30) to reach 3x RS7 seizures in the kindling period, whereas, in rekindling, it required an average of 5.25 ± 1.03 epochs to reach 3x RS7 (range: 4 to 6; *p* < 0.0001, paired Student’s *t*-test, Figure 3B). In the rekindling period, the first RS7 seizure was always followed by two consecutive RS7 seizures, our criteria for being fully kindled.

In summary, we demonstrated a successful kindling protocol by optogenetically activating glutamatergic neurons in the unilateral CA1 of the murine hippocampus. The effect of kindling was maintained even after the animals underwent a 14-day period of rest. No oPDs, oADs, or seizures were observed in sham (*n* = 6) or control animals (*n* = 4).

### Spatiotemporal changes of inhibitory signaling associated with OpK

We sought to evaluate whether our OpK protocol generated impairments in key elements of the inhibitory signaling of the hippocampus. We evaluated whether OpK in the CA1 layer was able to induce any global loss of parvalbumin-expressing interneurons (PV-INs) in the hippocampus. In addition, we evaluated the expression of the CCCs, i.e., KCC2 and NKCC1, the two main regulators of postsynaptic inhibition responsible for maintaining low [Cl^−^]i in neurons (Gagnon et al., 2013; Liu et al., 2019). A compromised inhibitory system is expected to manifest in a reduced number of PV-INs, a reduced KCC2 expression, and/or increased NKCC1 expression.

#### Reduced KCC2 in fully kindled and rekindled animals

**Figure 4:**
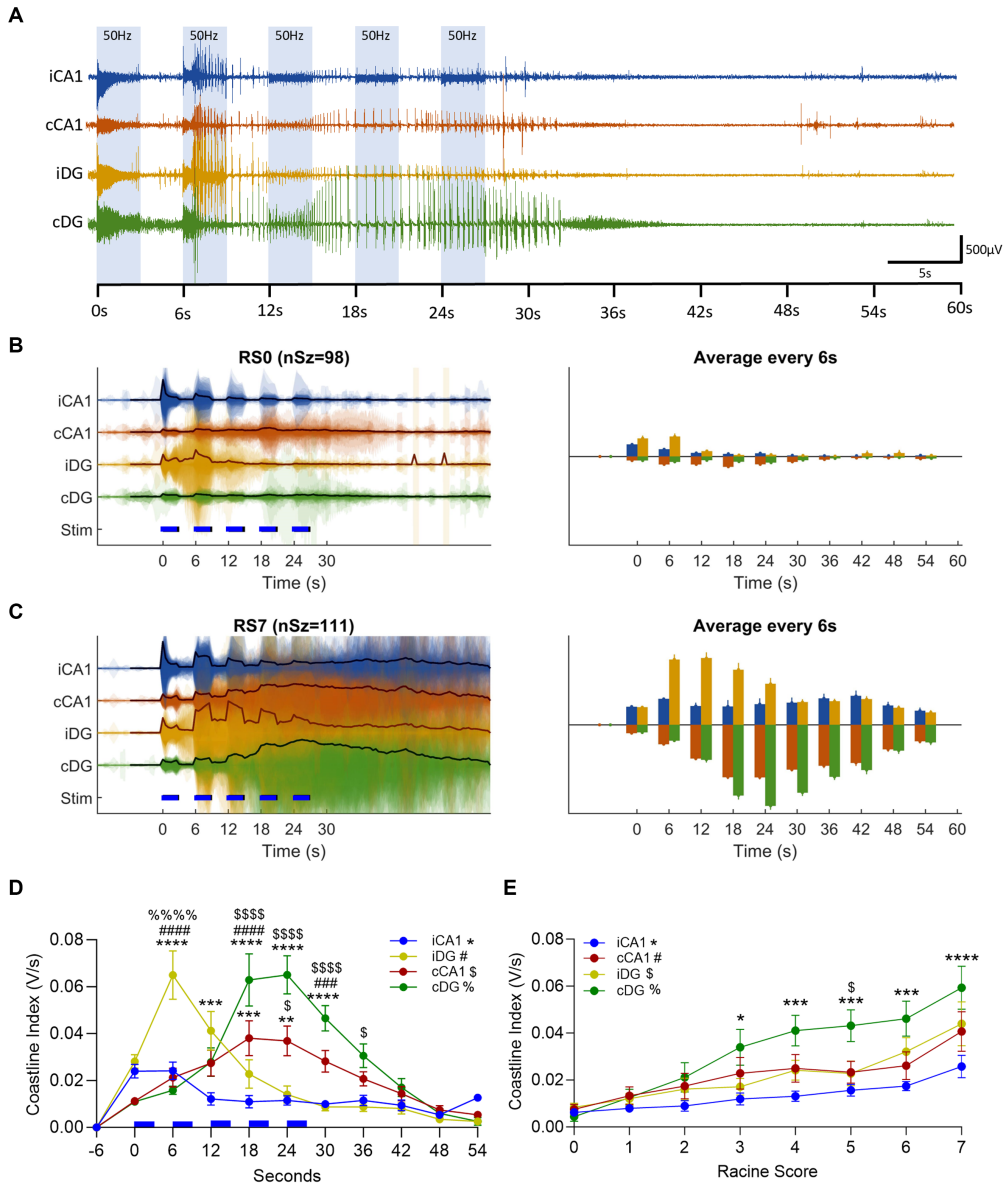
Optogenetic kindling disrupts the expression of the chloride co-transporters KCC2 and NKCC1. **(A)** Representative fluorescence photomicrographs of KCC2 and NKCC1 expression in the bilateral hippocampus of Sham, Ki, and Rek mice. Analyzed hippocampal sublayers from iHip (iCA1, iCA3, and iDG) and cHip (cCA1, cCA3, and cDG) are indicated in the upper row (Sham). Magnified photomicrographs of the DG layer are shown in the inserts a–l. **(B)** Averaged KCC2 Relative Protein Expression across Ki and Rek animals expressed as a percentage of the average level from Sham animals (Mean + SEM pooled across six 30 μm thick brain coronal sections from AP: ≅ −1.3 mm to ≅ −3.5 mm relative to Bregma; **p < 0.01; One-way ANOVA; with *post hoc* Dunnett’s multiple comparisons). Impairment in KCC2 expression was observed both in Ki and Rek animals. **(C)** As in panel **(B)** but for NKCC1. Upregulation of NKCC1 was observed in the hippocampus of Rek animals (*p < 0.05; One-way ANOVA; with *post hoc* Dunnett’s multiple comparisons). **(D)** Bar plot comparing the averaged KCC2 Relative Protein Expression of Ki and Rek animals grouped by hippocampal sublayers indicated in panel **(A)**. Note the downregulation of KCC2 was most pronounced in the cDG for the Ki and Rek groups, while in iCA1, where the laser stimulation was delivered, no significant KCC2 downregulation was observed (Mean + SEM pooled across all brain coronal sections as in panel **(B)**, relative to the corresponding average sublayer level from the Sham animals; *p < 0.05, **p < 0.01, ***p < 0.001; Two-way ANOVA; with *post hoc* Dunnett’s multiple comparisons test). **(E)** As in panel **(D)** but for NKCC1. Upregulations of NKCC1 expression were observed only in the iDG and cDG of Rek animals (*p < 0.05, **p < 0.01; Two-way ANOVA; with *post hoc* Dunnett’s multiple comparisons test).

In both hippocampi of fully kindled animals (Ki, *n* = 6), we observed a global decrease of 13.0% in KCC2 expression in comparison to the Sham group (Figures 4A,B, *p* < 0.01; Unpaired Student’s *t*-test). Although the laser stimulations were delivered to iCA1, greater decreases in KCC2 expression were observed in the contralateral hippocampus (cHip), rather than the ipsilateral hippocampus (iHip) (Table 1 and Figures 4A,D).

**Table 1:** Change in KCC2 and NKCC1 expression levels by hippocampal sublayer.

Hippocampal sublayer	KCC2		NKCC1	
	Ki	Rek	Ki	Rek
iCA1	−5.7%	−12.4%	−5.7%	13.4%
cCA1	−17.7%*	−14.8%*	−9.5%	4.1%
iCA3	−6.8%	−8.2%	2.6%	13.7%
cCA3	−16.9%*	−11.8%	0.3%	10.5%
iDG	−5.0%	−13.3%*	6.6%	24.1%*
cDG	−18.6%***	−13.7%**	7.4%	32.7%**

Values are expressed as relative percentage changes from the corresponding Sham group sublayer.

*p < 0.05, **p < 0.01, ***p < 0.001; Two-way ANOVA, with *post hoc* Dunnett’s multiple comparisons test.

Similar KCC2 expression levels were found in the rekindled animals (Rek, *n* = 8). Globally, KCC2 levels decreased by 11.9% in comparison to the Sham group (*n* = 6) (Figures 4A,B, *p* < 0.05; Unpaired Student’s *t*-test). A more pronounced decrease was found in the contralateral CA1 and DG (Table 1 and Figures 4A,D).

#### NKCC1 expression is increased only in chronically fully kindled mice

Unlike KCC2, NKCC1 showed no significant global alterations in the hippocampus of animals immediately after being fully kindled (Ki, *n* = 6) compared to Sham animals (*n* = 6) (Figures 4A,C; −2.4%, *p* = 0.9045; Unpaired Student’s *t*-test). No differences in the expression of NKCC1 were observed between hippocampal sublayers for the acute experimental group (Table 1 and Figures 4A,E).

Differently from the Ki animals, we observed a prominent global increase in NKCC1 expression in the hippocampus of animals of the Rek group (*n* = 8) (Figures 4A,C, Rek: 18.0%, *p* < 0.05, Unpaired Student’s *t*-test). The sublayer analysis in the chronic period revealed that NKCC1 increased in the DG bilaterally, with a stronger expression in the cDG (Table 1 and Figures 4A,E).

#### Loss of PV-INs was only observed in rekindled mice

**Figure 5:**
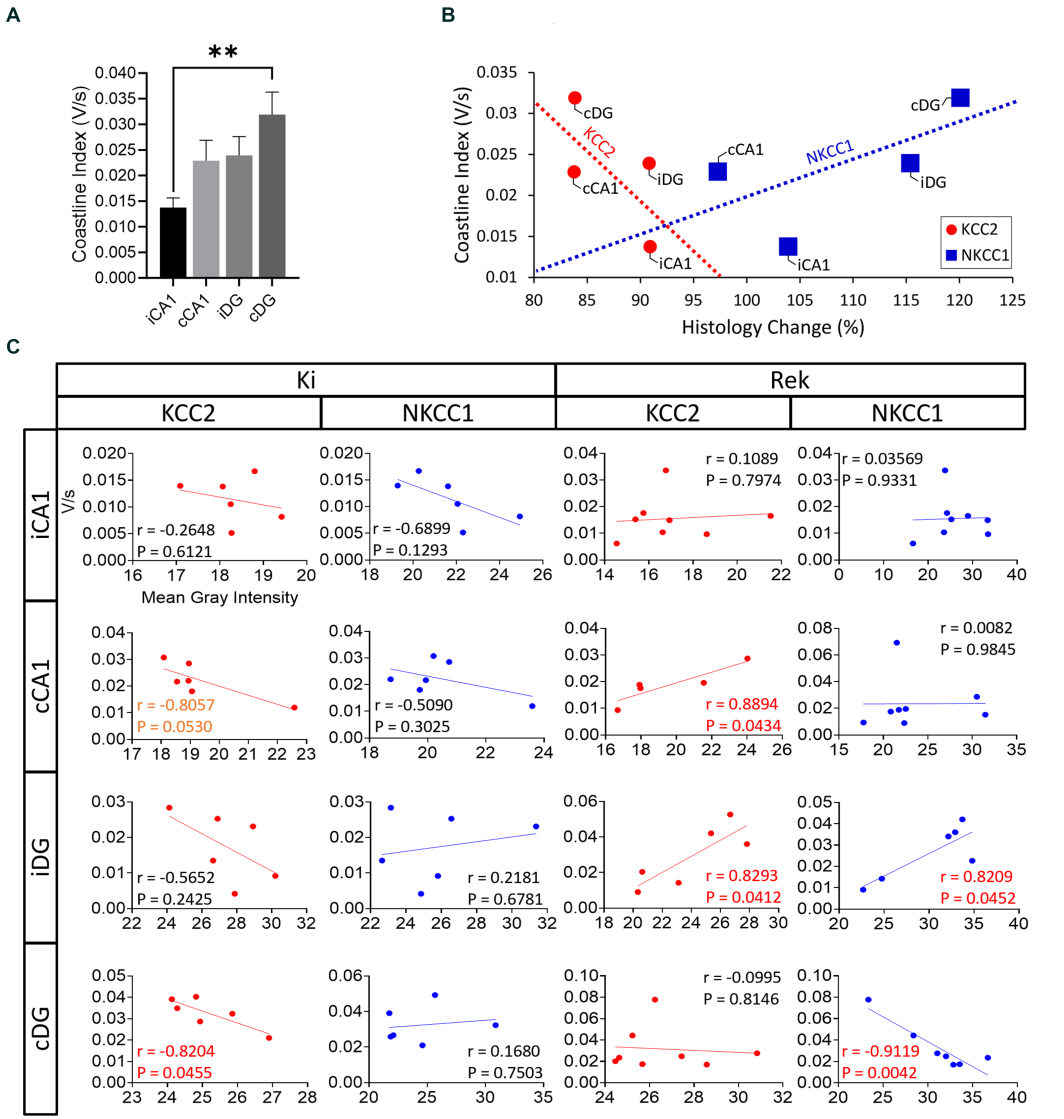
Parvalbumin-positive interneurons loss in the hippocampus of Rekindled animals. **(A)** Representative Parvalbumin-stained ventral hippocampus sections of mice perfused after being stimulated with dummy laser (Sham) and after rekindling stimulations (Rek). CA1, CA3, and DG sublayers are as indicated. Insets show magnified micrographs of the CA1 hippocampal sublayer indicated by boxes a and b of Sham and Rek, respectively. PV-INs are indicated by arrowheads in the inserts. **(B)** Average number of PV-INs for each mouse group (Mean + SEM). Counts were pooled bilaterally across six 30 μm thick coronal sections spanning AP: ≅ −1.3 mm to ≅ −3.5 mm relative to Bregma. PV-INs were reduced significantly in the hippocampus of rekindled animals (**p < 0.01; One-way ANOVA; with *post hoc* Dunnett’s multiple comparisons). **(C)** Average number of PV-INs in panel **(B)** separated by the side of the hippocampus (Mean + SEM). Note that there was no significant difference in the number of PV-INs between the iHip and cHip within each animal group (Two-way ANOVA; Tukey’s multiple comparisons as *post hoc* test). **(D)** Average number of PV-INs in panel **(B)** grouped by slice order from most anterior to most posterior (Mean + SEM). Approximated AP distance in mm from Bregma: Slice 1: 1.30 to 1.65, Slice 2: 1.65 to 2.00, Slice 3: 2.00 to 2.30, Slice 4: 2.30 to 2.65, Slice 5: 2.65 to 3.10, Slice 6: 3.10 to 3.50. Results are expressed in Average + SEM (**p < 0.01, ****p < 0.0001; Two-way ANOVA; Tukey’s multiple comparisons as *post hoc* test). Note that a reduction in the number of PV-INs was observed in the posterior (ventral) hippocampal sections of Ki and Rek animals.

We did not observe a significant reduction in the number of PV-INs in the hippocampi of animals from the Ki group (*n* = 6) in relation to the Sham animals (*n* = 6). The average number of PV-INs per brain slice in the Ki experimental group was 149.4 ± 8.4, while in the Sham group was 157.9 ± 13.9 (Figures 5A,B; *p* = 0.7467; Unpaired Student’s *t*-test). We did observe a decrease of 28.4% in the average number of PV-INs in the hippocampus of animals from the Rek group (*n* = 8) in comparison to the Sham group (*n* = 6). The average number of PV-INs per brain slice in the hippocampus of Rek animals was 113.0 ± 5.7 (Figures 5A,B; *p* < 0.01; Unpaired Student’s *t*-test). We then tested to see if there was a greater PV-INs loss at the side of optical stimulation. One could expect that the loss of PV-INS was greater at the site of optical stimulation. However, no statistically significant differences in the number of PV-INs were observed between the hippocampal sides within the experimental groups. In the hippocampus of Ki animals, the average number of PV-INs observed in the ipsilateral to the optical stimulation (iHip) was 77.0 ± 10.7, while the contralateral hippocampus (cHip) was 72.41 ± 12.9. In Rek animals, we observed an average number of PV-INs of 55.9 ± 8.5 in the iHip, while in the cHip it was 57.0 ± 7.7 (Figure 5C; *p* > 0.9999; Two-way ANOVA, with *post hoc* Tukey’s multiple comparisons test), indicating that the observed decrease in PV-IN counts is not specific to a hippocampal side.

We also tested to see if the brain slices close to the fiber cannula showed greater loss of PV-INs (Figure 5D). We did not observe any significant alteration in the number of PV-INs in slices in which the hippocampus was directly under the optic fiber in comparison to the respective slice number of the Sham group (Slices 2/3, Sham: 45.8 ± 14.9, Ki: 66.0 ± 21.3, Rek: 46.7 ± 17.5). While no overall loss of PV-INs was observed in animals from the Ki group, there were significant reductions in PV cells specifically in the posterior portion of the hippocampus. These reductions were observed in slices 5 (−20.9%) and 6 (−30.9%), which were located at distances of 0.52 and 0.98 millimeters, respectively, from the optic fiber placement slice (Slice 5 – Sham: 128.8 ± 36.0, Ki: 101.8 ± 25.7, *p* < 0.01; Slice 6 – Sham: 128.1 ± 35.4, Ki: 88.4 ± 23.5; *p* < 0.0001; Two-way ANOVA, with *post hoc* Tukey’s multiple comparisons test). In Rek animals, significant reductions in the number of PV-INs were observed in slice 5 (−46.5%) and slice 6 (−40.7%), located ~0.76 and ~ 1.07 millimeters more posterior to the optic fiber placement, respectively, (Slice 5 – Sham: 128.8 ± 36.0, Rek: 68.8 ± 15.7; Slice 6 – Sham: 128.1 ± 35.4, Rek: 75.9 ± 13.2; *p* < 0.0001, Two-way ANOVA, with *post hoc* Tukey’s multiple comparisons test). These findings suggest that neither heat generated during the laser stimulation, nor the immediate optically induced activity, were responsible for the loss of PV-IN cells.

### Distinct bilateral and layer-specific EEG activity patterns and aggravation over the course of OpK

**Figure 6:**
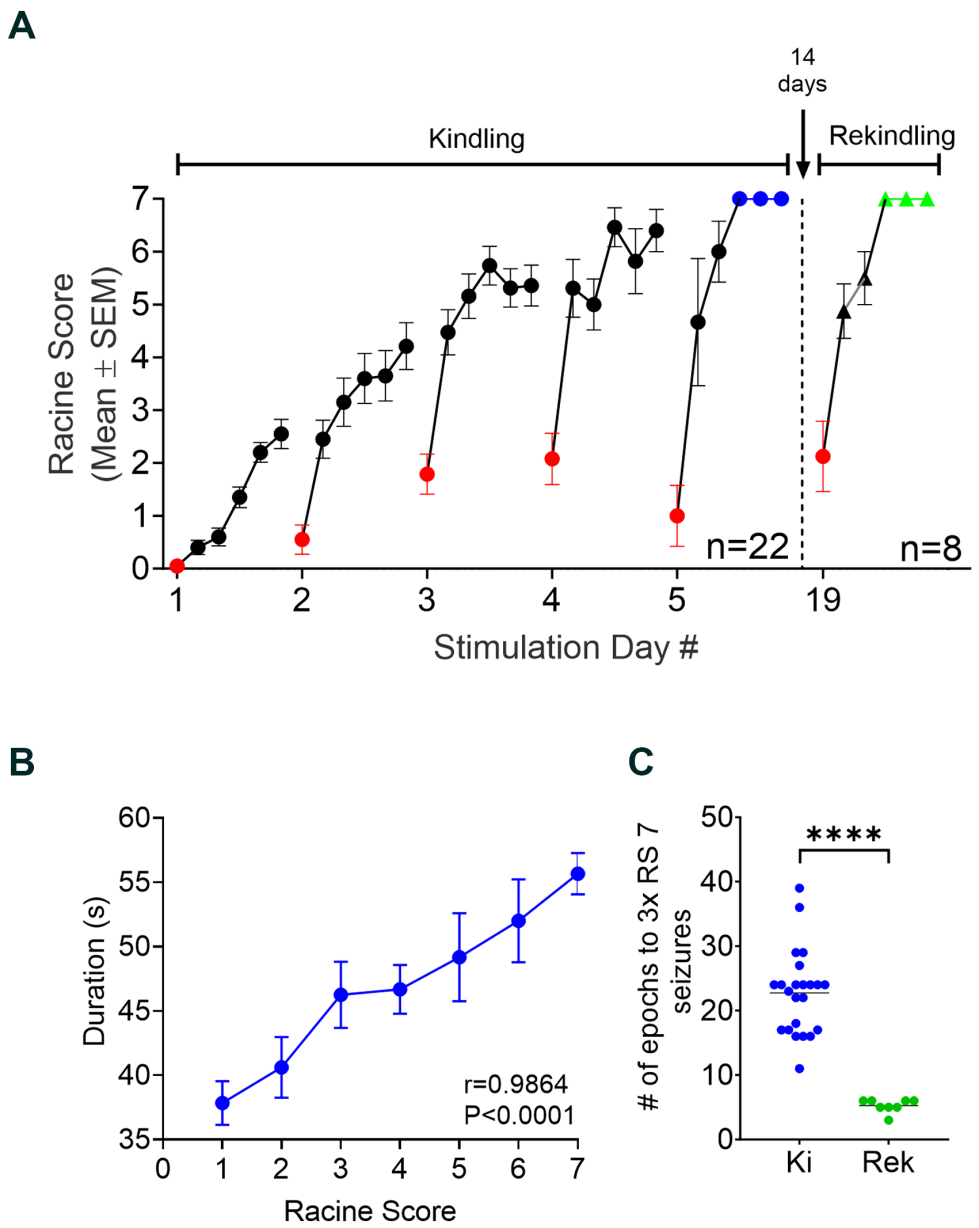
Optogenetic kindling in unilateral hippocampal CA1 revealed distinct seizure propagation to the contralateral hippocampus. **(A)** Representative epoch of a multichannel LFP recording for electrodes positioned in iCA1 (blue trace), cCA1 (red trace), iDG (yellow trace), and cDG (green trace). The ictal activity in display was evoked in response to the first stimulation epoch of the first day of stimulation of an animal and resulted in an RS0 seizure. Note that the first 3-s bout of 50 Hz stimulations elicited an immediate activation (oPDs) in all four channels, followed by spontaneous activity (oADs) that was sustained beyond the end of the epoch. **(B)** (left plot), Coastline index of the LFP activity for Racine score 0 seizures (RS0, nSz = 98). Colored patches represent the translucent overlay of the coastline index from all seizures, mirrored above and below the *x*-axis. The black line in each trace represents the mean coastline index. Blue dashed lines represent each 3 s-bout of laser stimulations. Right Plot – Averaged coastline index in 6 s windows (based on the 3 s-bout +3 s-interbout stimulation cycles). Upward and downward bars are used to better visualize sublayers and contrast iHip and cHip responses. (Upward bars: blue – iCA1, yellow – iDG, downward bars: red – cCA1, green – cDG). **(C)** as in panel **(B)** but for Racine score 7 seizures (RS7, nSz = 111 seizures). **(D)** Average coastline index across all seizures for each hippocampal sublayer (RS0 to RS7 in the kindling phase, nSz = 587, Mean ± SEM). Note that the initial activation of the iHip (iCA1 and iDG) is overpowered by the cHip activity around the fourth 6 s-cycle (18 s). Statistical testing was performed at each 6 s window across hippocampal sublayers (One-Way ANOVA); Tukey’s *post hoc* multiple comparison test was used to identify significantly different sublayer activity levels. Different symbols were used to represent significant differences with respect to each hippocampal sublayer: * – iCA1, # – iDG, $ – cCA1, % – cDG (*p < 0.05, **p < 0.01, ***p < 0.001, ****p < 0.0001). **(E)** Averaged coastline index across seizure severities (RS0 – RS7, Mean ± SEM) by hippocampal sublayer. The coastline index was averaged over 60s from the start of each epoch, then pooled across seizures from the same severity class. Note that cDG stands out from the other hippocampal sublayer with the progression of OpK. Statistical significances are denoted in the same way as in panel **(C)** (*p < 0.05, ***p < 0.001, ****p < 0.0001; One-Way ANOVA, with *post hoc* Tukey’s multiple comparison test).

The EEG response to the optical stimulation was characterized among different regions of the hippocampus by calculating the coastline index (CI) (Niknazar et al., 2013; Berglind et al., 2018). We observed an immediate neuronal activation time-locked with the laser pulses in all four recording channels (Figure 6A). The CI integrates the size of the step changes in the EEG signal and provides an effective way to quantify the level of EEG activity. Representing the EEG activity from early in the kindling process, in the non-behavior seizures (afterdischarges, RS0), we observed the smallest levels of CI activation (Figure 6B). On the continuous time-base (Figure 6B, left), the CI captured the step changes in the EEG activity between the stimulus-driven 3 s bouts and the stimulus-off inter-bouts, most pronounced in the iHip layers. CI from RS0 seizures showed a transient activation in iDG, peaking between *t* = 0–12 s, and otherwise very low activity levels. By contrast, the EEG activity level in RS7 seizures had increased several folds in RS7 seizures (Figure 6C) representing the EEG activity in fully-kindled animals. The activity level in RS7 seizures was most evident in the cHip layers. To better capture the overall trend, we integrate the CI over non-overlapping 6 s windows synchronized to the bout/inter-bout period (Figures 6B,C, right). This computational technique revealed distinctive activity patterns at different hippocampal sites (summarized in Figure 6D, grouped by RS level in Supplementary Figure 1); and distinctive patterns of electrophysiological power distribution as the seizure severity worsened over the course of OpK (Figure 6E).

We observed an immediate neuronal activation time-locked with the laser pulses in all four recording channels (Figures 6A–C). As the stimulation continued, the CI in the site of the optical stimulation (iCA1 and iDG) increased rapidly. The iDG channel displayed the earliest occurrence of oADs (Figures 6A–C, indicated by yellow traces). However, after the first stimulation bout/interbout (6 s), the averaged CI across seizures of different Racine scores (0–7) in iCA1 decreased and the EEG remained depressed even during the ictal activity (Figure 6D, blue line). With the emergence of oADs, the CI of iDG surpassed that of iCA1 between 6 and 12 s after the optical stimulation started (Figure 6D; *p* < 0.01, One-Way ANOVA, with *post hoc* Tukey’s multiple comparison test). In iDG, the CI peaked at 6.0 ± 3.8 s after the optical stimulation started and gradually decayed afterward, remaining relatively quiet during ictal activity. By contrast, the CI measurements of the contralateral channels (cCA1 and cDG, Figures 6A–C, red and green traces, respectively) gradually increased until peaking at 21.0 ± 4.2 s after the start of stimulation. The cDG was the most active channel and exhibited a CI on average 76% higher than cCA1 (*p* < 0.01), 367% higher than iDG (*p* < 0.0001), and 461% higher than iCA1 (Figure 6D; *p* < 0.0001, One Way ANOVA, with *post hoc* Tukey’s multiple comparison test). The average time when the cHip EEG activity overpowered iHip occurred 18 ± 3 s after the start of the optical stimulation (Figure 6D). We observed this overpowering in the EEG activity from non-behavioral seizures (RS0), and more evidently in behavioral seizures (RS1-7, Supplementary Figure 1). The cHip overpowering timing coincides with the onset of the behavioral component (18 ± 1 s, *n* = 22). We also observed an increase in the CI corresponding to the worsening of the seizure severity across the progression of kindling (Figure 6E). The cDG displayed a gradual increase in the CI across the seizures of different Racine scores when compared to the other hippocampal sublayers, in particular iCA1 (Figure 6E – RS3: cDG vs. iCA1, *p* < 0.05, RS4: cDG vs. iCA1, *p* < 0.001; RS5: cDG vs. iCA1, *p* < 0.001, cDG vs. iDG, *p* < 0.05; RS6: cDG vs. iCA1, *p* < 0.001; RS7: cDG vs. iCA1, *p* < 0.0001; One Way ANOVA, with *post hoc* Tukey’s multiple comparison test).

### Correlation between EEG activity aggravation and inhibitory impairment

**Figure 7:**
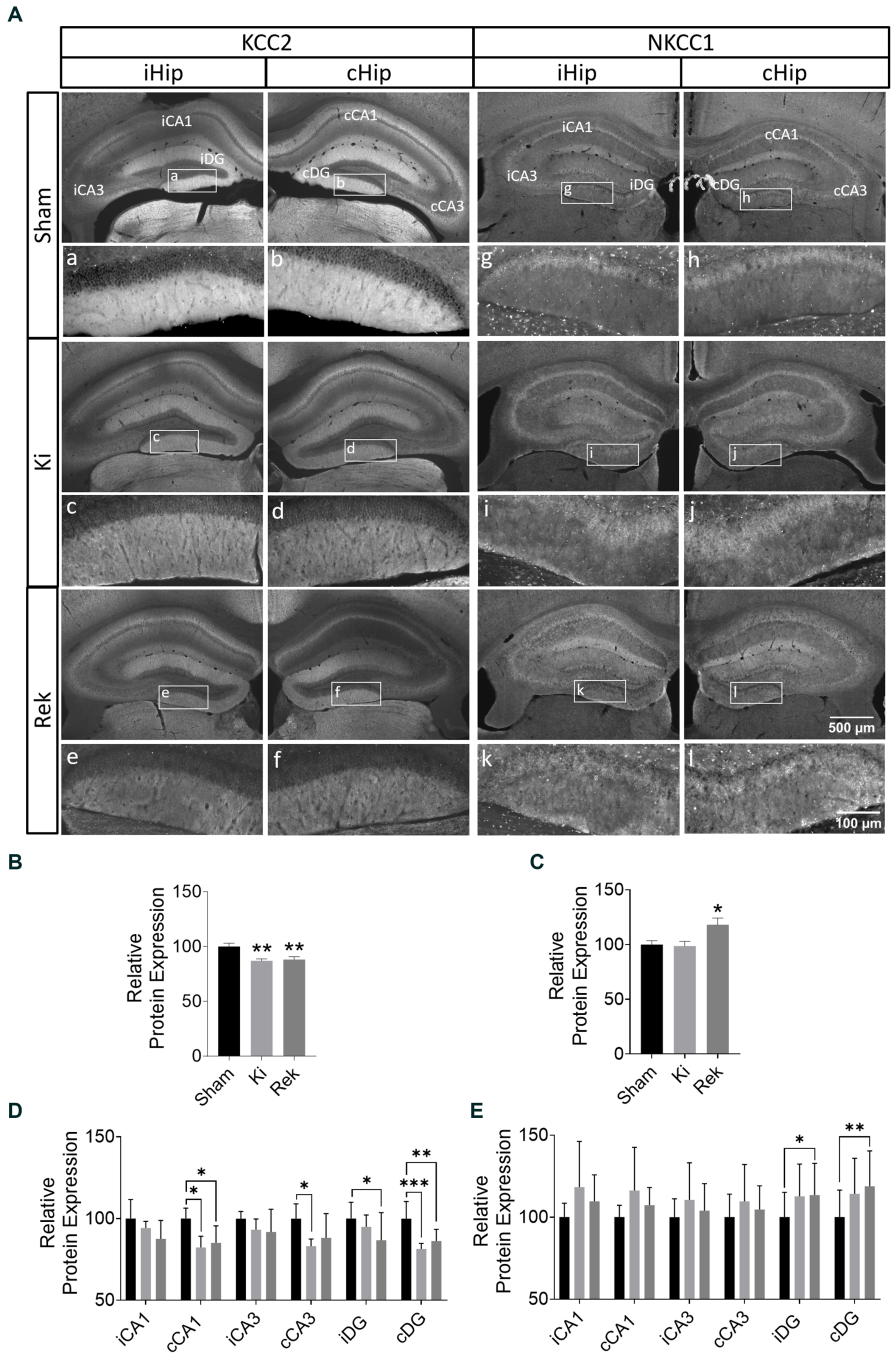
Correlation analysis between KCC2 and NKCC1 impairments and seizure propagation pattern. **(A)** Averaged coastline index by hippocampal sublayer pooled across all mice (*n* = 22) in the entire experiment (Mean + SEM). cDG showed the greatest overall activation (**p < 0.01 in comparison to iCA1, Student’s *t*-test). **(B)** Correlation analysis between averaged relative protein expression (histology change – %), and the averaged coastline index, where all the red circles and red dashed line correspond to KCC2 and blue the blue squares and blue dashed line correspond to NKCC1. Note that greater levels of coastline index were generally associated with greater levels of protein impairment; however, our results did not reach statistical significance (KCC2: *r* = −0.6654, *p* = 0.3346; NKCC1: *r* = 0.6577, *p* = 0.3423, Two-tailed Pearson r correlation test). **(C)** Correlation analysis between the averaged RFI obtained from the expression of each protein analyzed (KCC2 or NKCC1) grouped by hippocampal sublayers and by experimental period (Ki or Rek). Correlation (r) and *p* values are indicated in each plot; *p* < 0.1 correlations in orange text, *p* < 0.05 correlations in red text.

Impairment of the inhibitory function has been suggested to be a leading factor behind kindling (Kamphuis et al., 1991). Here, we observed that the OpK model induced inhibitory impairments with different temporal and regional alterations in the expression of KCC2 and NKCC1 in bilateral hippocampi. We sought to consider whether the regional alterations in KCC2 and NKCC1 expression may be correlated to the regional EEG power measured using the CI. We examined this relationship on two levels: (i) at the network level, in which we reviewed the overall association between EEG CI and KCC2/NKCC1 expression (Figure 7B), and (ii) at the individual animal level, where we conducted a correlation analysis to examine the relationship between the EEG CI and the expressions of KCC2/NKKC1 in the respective hippocampal sublayers of animals in both experimental groups (Figure 7C).

To summarize the regional EEG power increase observed in Figure 6D, the CI for each hippocampal sublayer was integrated across all seizures within mice (Figure 7A, values expressed in averaged CI). At the network level, the average o EEG CI value across all mice was plotted against the corresponding average regional changes in KCC2 and NKCC1 expressions across mice (Figures 4D,E). Although no significant correlation was found at the network level, two interesting trends were observed (Figure 7B; KCC2: *r* = −0.66, *p* = 0.33, red dashed line; NKCC1: *r* = 0.65, *p* = 0.34, blue dashed line; Two-tailed Pearson r correlation test). For KCC2, an overall negative trend was observed. The cDG, which demonstrated greater CI values in response to the repetitive optical stimulation, showed the lowest relative levels of KCC2. The low relative levels of KCC2 expression observed in the cDG were also seen in the cCA1, however, cCA1 required lower levels of activation (Figure 7B). In contrast, in iCA1, the hippocampal sublayer with overall smaller CI values had the lower degree of KCC2 impairments (see Figure 5D). For NKCC1, the hippocampal sublayers with greater CI values were associated with higher levels of NKCC1 expression, and *vice-versa*.

We further examined these trends at the individual animal level using correlation analysis and found a mixture of regional-specific trends and differences between Ki and Rek experiment groups. For KCC2, regional trends in the Ki animals were consistent with the negatively correlated network-level trend (cCA1: *r* = −0.80, *p* = 0.05; cDG: *r* = −0.82, *p* = 0.04; Two-tailed Pearson’s r correlation test). In the mice that underwent the rekindling process, a dramatic change in the correlation between KCC2/NKCC1 levels and EEG CI was observed. In these animals, the relationship between KCC2 and CI values was reversed, whereas we observed a significant positive correlation in cCA1 and iDG (cCA1: *r* = 0.88, *p* = 0.04; iDG: *r* = 0.82, *p* = 0.04; Two-tailed Pearson’s r correlation test). For NKCC1, against the network-level trend, CI values negatively correlated in Ki animals (iCA1 and cCA1). In Rek animals, opposite trends with significant correlation were observed in iDG and cDG sublayers (iDG: *r* = 0.82, *p* = 0.04; cDG: *r* = −0.91, *p* = 0.004) (Figure 7C).

## Discussion

In this study, we implemented an OpK model in freely moving mice by repeatedly activating putative pyramidal neurons in the CA1 region of the unilateral hippocampus, the most vulnerable hippocampal area in patients with TLE (Wittner et al., 2005). We observed impairments in KCC2 and NKCC1 expression, with distinctive spatiotemporal changes in the mice hippocampi (Figure 4), as well as loss of PV-INs in the hippocampi of animals that had undergone rekindling (Figure 5). We also identified a distinct neural activation sequence between iHip and cHip, which had specific associations with KCC2 and NKCC1 impairment patterns. The findings shown here may provide insight into the cellular and molecular changes in the inhibitory signaling underlying the kindling phenomenon observed among patients with medically intractable epilepsy.

### KCC2 is the first inhibitory component to become impaired after kindling

Chloride is the most abundant physiological anion and is fundamental for inhibitory GABAergic signaling in neurons (Auer et al., 2020). Neuronal [Cl^−^]i homeostasis ensures properly functioning of the inhibitory signaling and KCC2 is a main regulator for maintaining [Cl^−^]i homeostasis in mature neurons (Liu et al., 2019). Our observations showing that KCC2 is downregulated in all analyzed sublayers of the cHip of fully kindled animals contradicts the results from a previous report (Rivera et al., 2002). In their study, Rivera et al. showed that downregulations in KCC2 expression were observed in both iHip and cHip in response to EK. It is possible that additional ipsilateral changes observed as a result of EK may be due to the lack of cell-type specificity of electrical stimulation (Cela et al., 2019). The optical stimulations employed in our experiments selectively targeted putative pyramidal neurons. Another hypothesis is that the focal optical stimulations in unilateral CA1 may have allowed the triggering of an innate feedback inhibitory signaling that DG performs onto CA hippocampal areas in response to ictal activity (Gutierrez and Heinemann, 2001). Therefore, this inhibitory circuit may operate as a preventing mechanism against seizure-dependent cellular damage, consequently maintaining normal levels of KCC2.

Previous studies have suggested that impairments in KCC2 expression, in addition to being a result of recurrence of seizure activity, it may also be a contributing factor to the seizure intensification across the progression of the kindling (Chen et al., 2017). This suggests that the altered KCC2 expression observed here may be contributing to the aggravation of seizures. Disturbed [Cl^−^]i levels resulting from reduced expression of KCC2 may create a propitious environment for pathological seizure propagation (Sloviter, 1994; Doyon et al., 2015). The downregulation of KCC2 observed in the cHip of fully kindled animals may thus have compromised the neuronal responsiveness to inhibitory GABAergic signaling, which imply in the development of a gradual chloride-dependent networkwide disinhibition, progressively “steering” the recruitment of new neural clusters into more seizure-susceptible ones. As a result, a preferential augmentation of EEG power in the cHip, specifically in cDG was observed across the progression of kindling.

### Chronic alterations in NKCC1 expression

In contrast to the early changes in KCC2, our study only found NKCC1 expression to be upregulated in animals that had undergone rekindling. Although the late NKCC1 regulation observed here may have an association with the stimulations delivered in the rekindling phase, it is unlikely that it is caused solely by it. Animals from this experimental group were sacrificed at least 14 days after the animals from the Ki group. For his reason, we speculate that the hippocampi of these animals underwent a continuous self-modifying process after becoming fully kindled, that might have been amplified by the seizures evoked in the rekindling period. Additionally, the late NKCC1 upregulation observed here contradicts the findings of previous studies that showed NKCC1 upregulated acutely following EK (Okabe et al., 2003), kainic acid-induced status epilepticus (KA-SE) (Nogueira et al., 2015), and pilocarpine-induced SE (Brandt et al., 2010). A hypothesis to explain this result is that these animals may have developed reactive astrogliosis (Khurgel et al., 1995; Cole-Edwards et al., 2006). This hypothesis is based on the premise that, unlike KCC2, which is expressed exclusively in neurons (Papp et al., 2008; Moore et al., 2017; Herrmann et al., 2021), NKCC1 is also largely present in astrocytes (Khirug et al., 2008; Löscher et al., 2013; Virtanen et al., 2020). Extensive data has shown that the animal models of epilepsy mentioned earlier rapidly develops reactive astrogliosis in the brain, either directly, as in the KA-SE model (Vargas et al., 2013), or indirectly, as the pilocarpine-induced SE (Shapiro et al., 2008), and in EK (Khurgel et al., 1995). Even though we did not evaluate the presence of reactive astrocytes in our study, another study has shown that fully kindled animals that underwent a similar OpK protocol did not develop reactive astrogliosis (Cela et al., 2019). Another possibility is NKCC1 upregulation being a result of the maintenance of the low [Cl^−^]i in neurons and extracellular potassium ([K^+^]_o_) accumulation, which may be caused by persistent downregulation of KCC2 during epileptogenesis (Frohlich et al., 2008; Pallud et al., 2014; Raimondo et al., 2015). Therefore, our results support the idea that NKCC1 upregulation is the endpoint result of a seizure-dependent facilitation resulting from the kindling process. We also speculate that NKCC1 upregulation, in addition to KCC2 downregulation, is the molecular mechanism behind the increased seizure susceptibility observed in rekindled animals, once changes in the expression of these two CCCs are described to result in dysfunctional GABAergic inhibitory capabilities, thereafter resulting in increased cellular excitability (Sen et al., 2007).

### Loss of parvalbumin-positive interneurons is observed bilaterally in the hippocampus of rekindled animals

PV-INs are one of the main regulators of excitation/inhibition (E/I) balance and timing of excitatory neurons firing by performing perisomatic GABAergic inhibition of excitatory neurons in the brain (Godoy et al., 2022). We showed that the number of PV-INs significantly reduced in both iHip and cHip of animals that underwent rekindling. The loss of PV-INs likely occurs during the epileptogenesis process (Huusko et al., 2015), and is implicated with increased excitability in epileptic states, being considered a hallmark of TLE in humans (Sloviter et al., 1991; Godoy et al., 2022), and animal models (Kuruba et al., 2011; Löscher, 2011; Pitkanen et al., 2015). Our results indicate that the OpK model induces some level of epileptogenesis, and the loss of PV-INs from OpK can be more severe and extensive compared to previous reports which found only mild PV-INs loss ipsilaterally (Ryu et al., 2021). The chronic loss of PV-INs may also be associated with the chronic disruption of KCC2, via mitogen-activated protein kinase- (MAPK) dependent apoptosis (Herrmann et al., 2021).

### OpK progression revealed a distinct pattern of seizure propagation throughout the bilateral hippocampi

The kindling animal model, traditionally induced with electrical stimulations, has been an important tool in investigating the neurobiology of the kindling phenomenon observed among patients with medically intractable epilepsy. The development of optogenetics allowed other groups to successfully develop OpK models targeting other brain regions of animals (Cela et al., 2019; Klorig et al., 2019; Butler et al., 2020; Ryu et al., 2021; Shimoda et al., 2022), which inspired us to develop the OpK model employed in this study. To the best of our knowledge, our OpK model is the first protocol targeting the dorsal hippocampal CA1 layer unilaterally. As a proof of principle, our OpK model reproduced the main characteristics observed in classical EK models, such as progressive intensification in seizure severity and duration, and long-term retention of the kindling effect once the animal is fully kindled (McNamara, 1986; Bertram, 2007).

One distinct advantage of the OpK approach over EK is that optical stimulations do not produce electrical artifacts in the EEG during stimulation (Ryu et al., 2021). This allowed us to capture and examine the immediate EEG response to optical stimulation pulses which are otherwise masked in EK. The analysis of the evoked responses revealed an immediate hippocampal activation in all recording channels time-locked with the laser pulses (oPDs, Figure 2C, insert a; Figure 6A). Predominantly, oPDs were followed by the emergence of ictal-like discharges (oADs; Figure 2C, insert b; Figure 6A), which is consistent with a similar study (Berglind et al., 2018).

We observed that the different sublayers of the bilateral hippocampus displayed different levels of CI within each stimulation epoch as result of the focal activation of CA1, revealing a distinct pattern of seizure propagation throughout the hippocampus. To our knowledge, this is the first study to quantify the changes in EEG patterns during the process of kindling using CI. The transient activation/deactivation of EEG in the iHip observed in response to the optical stimulation is surprising. One might expect that the oAD activity in the iHip would continually increase in response to the optical stimulations since this region is the presumed seizure onset zone, and therefore the source of ictal EEG activity during the seizure. Yet, our analyses showed a drastic decrease in activity levels in iHip, followed by a transference of this activity to cHip, which exhibited higher levels of EEG activity still during the length of optical stimulation. This deactivation of the iHip observed in our experiments suggests that the optical stimulation may also have triggered a complementary inhibitory response in the iHip that is otherwise not observed in the cHip. Three possible forms of inhibitory response have been identified to explain this phenomenon: (i) a local CA1 GABAergic feedback transmission (Esclapez et al., 1997); (ii) the reduction in iHip activity can emerge from an activity-dependent transient fast inhibition mechanism from DG to CA3 via GABAergic mossy fiber projections (Gutierrez and Heinemann, 2001); and (iii) a DG-mediated inhibitory signaling through reentrant signals (Pare et al., 1992). Because of the temporal lobe anatomical properties, epileptiform activity originating in CA1 extends unidirectionally toward the subiculum through extrahippocampal areas (Orman et al., 2008). As such, ictal activity evoked in CA1 is more likely to propagate to extrahippocampal areas and return to the hippocampus via reentrant loops to DG (Pare et al., 1992). Therefore, once the DG is believed to be the hippocampal inhibitory gateway to extrahippocampal epileptiform signaling (Krook-Magnuson et al., 2015), impairments in inhibitory signaling elements, such as KCC2 and NKCC1, may make the DG more susceptible to ictal activity, possibly exacerbating the development of seizures.

### Physiologic implications: kindling and EGABA

The upregulation of NKCC1 expression, expansion of KCC2 downregulation, and reduction in PV-INs observed in the rekindled animals suggest that the hippocampal networks continued to undergo self-modifications during the 14-day rest period after the completion of kindling, which is consistent with the notion of epileptogenesis (Löscher et al., 2013; Liu et al., 2019).

Alterations in the expression of these two CCCs may generate an excessive accumulation of [Cl^−^]i and increased [K^+^]o. Increased [Cl^−^]i and [K^+^]o results in the shift of the GABA_A_R-mediated postsynaptic potentials (EGABA) by the generation of an inwardly-directed electrical depolarizing GABAergic effect (Pathak et al., 2007; Viitanen et al., 2010; Doyon et al., 2015; Liu et al., 2019). This shift may turn the normal GABAergic feedback inhibition into a positive ictogenic feedback loop (Kahle et al., 2016), and result in network overexcitation, increasing neuronal susceptibility to abrupt transitions from interictal to ictal states (Pathak et al., 2007; Sen et al., 2007; Kahle et al., 2016; Liu et al., 2019; Herrmann et al., 2021). This mechanism is also described to be the biochemical basis of a disinhibitory GABA-mediated neurotransmission that contributes to the increased seizure susceptibility observed in patients with epilepsy (Kamphuis et al., 1991; Cobb et al., 1995; Nusser et al., 1998; Dzhala et al., 2005; Kahle et al., 2016).

### Clinical implications: kindling and mirror focus

Understanding the inhibitory mechanisms underlying the promotion of the kindling phenomenon in TLE is essential to developing new approaches against epilepsy. One intriguing finding of our study is that impairment of inhibitory signaling components was more severe in the cHip, where neurons were not directly activated by the optical stimulations. This result is likely due to a transference phenomenon of the ictal activity to the contralateral hippocampus, in which cDG was the area that displayed the most severe EEG activity over the course of kindling. We believe that our OpK model led to the precursor of a process known as secondary epileptogenesis, where recurrent spread and generalization of hypersynchronous epileptiform discharges lead to the recruitment of neuronal clusters outside the primary seizure focus (Morrell et al., 1975; Salanova et al., 1994; Ben-Ari and Dudek, 2010; Gollwitzer et al., 2017; Bjellvi et al., 2019; Liu et al., 2020; Shen et al., 2021). Secondary epileptogenesis can ultimately lead to a transference of epileptogenicity to the homotopic area contralateral to the primary focus, resulting in the development of mirror foci (Sato, 1976; Sloviter, 1994). Mirror foci have been reported in several epilepsy models in different animal species (Wilder et al., 1968; Morrell et al., 1975; Tsuru, 1981), as in fully kindled cats, whereas generalized seizures were promptly evoked by challenging the contralateral hippocampus with electrical stimulation with the same parameters as applied to the primary (ipsi) focus (Sato, 1976).

Mirror foci have also been reported in patients with epilepsy (Schmidt et al., 2004; Gollwitzer et al., 2017). Epileptic patients with detected mirror foci, on average show a decreased probability of achieving seizure freedom after epilepsy surgery (Salanova et al., 1994; Yoon et al., 2003; Ben-Ari and Dudek, 2010; de Tisi et al., 2011; Gollwitzer et al., 2017; Bjellvi et al., 2019). Poor surgical outcomes have been associated with the development of a kindling process in patients with longer epilepsy as the preoperative time increases (Salanova et al., 1994; Luders, 2001; Kim et al., 2014; Park et al., 2018; Liu et al., 2020). The generation of mirror foci in patients with epilepsy is also associated with pharmacoresistance to conventional ASDs (Bernasconi et al., 2005; Shen et al., 2021). It is suggested that chronic disruptions in NKCC1 expression and function may alter the function of conventional drug targets, such as GABA-A receptors, contributing to pharmacoresistance (Aronica et al., 2007; Sen et al., 2007). Indeed, increased expression of GABA-A receptors was found in the mirror focus of a patient with long-term epilepsy (Bortolato et al., 2010), reinforcing the role of EGABA in the initiation and propagation of epileptic activity (Barmashenko et al., 2011; Khazipov et al., 2015). This suggests that the highest level of EEG activity during seizures from OpK found in the cHip, in addition to dysfunctions in the inhibitory signaling of the homotopic area contralateral to the primary focus, as observed in our study, may play a large role in the formation of mirror foci. Furthermore, the OpK model may provide a valuable tool for further examining the role of inhibitory signaling in preventing the formation of mirror as a consequence of secondary epileptogenesis and may offer a potential explanation for the increased pharmacoresistance observed in patients with mirror foci.

## Data availability statement

The original contributions presented in the study are included in the article/Supplementary material, further inquiries can be directed to the corresponding author.

## Ethics statement

The animal study was approved by Rutgers Institutional Animal Care and Use Committee (IACUC). The study was conducted in accordance with the local legislation and institutional requirements.

## Author contributions

FT: Conceptualization, Formal analysis, Investigation, Methodology, Supervision, Validation, Visualization, Writing – original draft, Writing – review & editing. DV: Writing – review & editing. SC: Conceptualization, Data curation, Formal analysis, Investigation, Methodology, Supervision, Validation, Writing – review & editing. HS: Conceptualization, Data curation, Formal analysis, Project administration, Resources, Supervision, Validation, Writing – review & editing.

## Footnotes

^1^ https://web.stanford.edu/group/dlab/optogenetics/

## References

[ref1] Achilles K. Okabe A. Ikeda M. Shimizu-Okabe C. Yamada J. Fukuda A.. (2007). Kinetic properties of Cl uptake mediated by Na+−dependent K+-2Cl cotransport in immature rat neocortical neurons. J. Neurosci. 27, 8616–8627. doi: 10.1523/JNEUROSCI.5041-06.2007, PMID: 17687039 PMC6672936

[ref2] Aravanis A. M. Wang L. P. Zhang F. Meltzer L. A. Mogri M. Z. Schneider M. B.. (2007). An optical neural interface: in vivo control of rodent motor cortex with integrated fiberoptic and optogenetic technology. J. Neural Eng. 4, S143–S156. doi: 10.1088/1741-2560/4/3/S02 17873414

[ref3] Aronica E. Boer K. Redeker S. Spliet W. G. Van Rijen P. C. Troost D.. (2007). Differential expression patterns of chloride transporters, Na+-K+-2Cl--cotransporter and K+-cl--cotransporter, in epilepsy-associated malformations of cortical development. Neuroscience 145, 185–196. doi: 10.1016/j.neuroscience.2006.11.041, PMID: 17207578

[ref4] Auer T. Schreppel P. Erker T. Schwarzer C. (2020). Impaired chloride homeostasis in epilepsy: molecular basis, impact on treatment, and current treatment approaches. Pharmacol. Ther. 205:107422. doi: 10.1016/j.pharmthera.2019.107422, PMID: 31626872

[ref5] Barmashenko G. Hefft S. Aertsen A. Kirschstein T. Kohling R. (2011). Positive shifts of the Gabaa receptor reversal potential due to altered chloride homeostasis are widespread after status epilepticus. Epilepsia 52, 1570–1578. doi: 10.1111/j.1528-1167.2011.03247.x, PMID: 21899534

[ref6] Ben-Ari Y. Dudek F. E. (2010). Primary and secondary mechanisms of epileptogenesis in the temporal lobe: there is a before and an after. Epilepsy Curr. 10, 118–125. doi: 10.1111/j.1535-7511.2010.01376.x, PMID: 20944823 PMC2951692

[ref7] Berglind F. Andersson M. Kokaia M. (2018). Dynamic interaction of local and transhemispheric networks is necessary for progressive intensification of hippocampal seizures. Sci. Rep. 8:5669. doi: 10.1038/s41598-018-23659-x, PMID: 29618778 PMC5884800

[ref8] Bernasconi N. Natsume J. Bernasconi A. (2005). Progression in temporal lobe epilepsy: differential atrophy in mesial temporal structures. Neurology 65, 223–228. doi: 10.1212/01.wnl.0000169066.46912.fa 16043790

[ref9] Bertram E. (2007). The relevance of kindling for human epilepsy. Epilepsia 48, 65–74. doi: 10.1111/j.1528-1167.2007.01068.x 17571354

[ref10] Bjellvi J. Olsson I. Malmgren K. Wilbe Ramsay K. (2019). Epilepsy duration and seizure outcome in epilepsy surgery: a systematic review and meta-analysis. Neurology 93, e159–e166. doi: 10.1212/WNL.0000000000007753, PMID: 31182508 PMC6656653

[ref11] Blumenfeld H. Varghese G. I. Purcaro M. J. Motelow J. E. Enev M. Mcnally K. A.. (2009). Cortical and subcortical networks in human secondarily generalized tonic-clonic seizures. Brain 132, 999–1012. doi: 10.1093/brain/awp028, PMID: 19339252 PMC2724910

[ref12] Bonansco C. Fuenzalida M. (2016). Plasticity of hippocampal excitatory-inhibitory balance: missing the synaptic control in the epileptic brain. Neural Plast. 2016:8607038. doi: 10.1155/2016/8607038 27006834 PMC4783563

[ref13] Bortolato M. Barberini L. Puligheddu M. Muroni A. Maleci A. Ennas F.. (2010). Involvement of Gaba in mirror focus: a case report. Epilepsy Res. 90, 300–303. doi: 10.1016/j.eplepsyres.2010.05.012, PMID: 20558041

[ref14] Brandt C. Nozadze M. Heuchert N. Rattka M. Löscher W. (2010). Disease-modifying effects of phenobarbital and the Nkcc1 inhibitor bumetanide in the pilocarpine model of temporal lobe epilepsy. J. Neurosci. 30, 8602–8612. doi: 10.1523/JNEUROSCI.0633-10.2010, PMID: 20573906 PMC6634618

[ref15] Butler C. R. Boychuk J. A. Pomerleau F. Alcala R. Huettl P. Ai Y.. (2020). Modulation of epileptogenesis: a paradigm for the integration of enzyme-based microelectrode arrays and optogenetics. Epilepsy Res. 159:106244. doi: 10.1016/j.eplepsyres.2019.106244, PMID: 31816591 PMC7451242

[ref16] Cela E. Mcfarlan A. R. Chung A. J. Wang T. Chierzi S. Murai K. K.. (2019). An optogenetic kindling model of neocortical epilepsy. Sci. Rep. 9:5236. doi: 10.1038/s41598-019-41533-2 30918286 PMC6437216

[ref17] Cela E. Sjostrom P. J. (2019). Novel optogenetic approaches in epilepsy research. Front. Neurosci. 13:947. doi: 10.3389/fnins.2019.00947, PMID: 31551699 PMC6743373

[ref18] Chen L. Wan L. Wu Z. Ren W. Huang Y. Qian B.. (2017). Kcc2 downregulation facilitates epileptic seizures. Sci. Rep. 7:156. doi: 10.1038/s41598-017-00196-7, PMID: 28279020 PMC5427808

[ref19] Choy M. Dadgar-Kiani E. Cron G. O. Duffy B. A. Schmid F. Edelman B. J.. (2021). Repeated hippocampal seizures lead to brain-wide reorganization of circuits and seizure propagation pathways. Neuron 110, 221–236.e4. doi: 10.1016/j.neuron.2021.10.010 34706219 PMC10402913

[ref20] Cobb S. R. Buhl E. H. Halasy K. Paulsen O. Somogyi P. (1995). Synchronization of neuronal activity in hippocampus by individual gabaergic interneurons. Nature 378, 75–78. doi: 10.1038/378075a0, PMID: 7477292

[ref21] Cole-Edwards K. K. Musto A. E. Bazan N. G. (2006). C-Jun N-terminal kinase activation responses induced by hippocampal kindling are mediated by reactive astrocytes. J. Neurosci. 26, 8295–8304. doi: 10.1523/JNEUROSCI.1986-05.2006, PMID: 16899724 PMC6673801

[ref22] Cossart R. Dinocourt C. Hirsch J. C. Merchan-Perez A. De Felipe J. Ben-Ari Y.. (2001). Dendritic but not somatic Gabaergic inhibition is decreased in experimental epilepsy. Nat. Neurosci. 4, 52–62. doi: 10.1038/82900 11135645

[ref23] De Lanerolle N. C. Kim J. H. Robbins R. J. Spencer D. D. (1989). Hippocampal interneuron loss and plasticity in human temporal lobe epilepsy. Brain Res. 495, 387–395. doi: 10.1016/0006-8993(89)90234-5, PMID: 2569920

[ref24] De Tisi J. Bell G. S. Peacock J. L. Mcevoy A. W. Harkness W. F. Sander J. W.. (2011). The long-term outcome of adult epilepsy surgery, patterns of seizure remission, and relapse: a cohort study. Lancet 378, 1388–1395. doi: 10.1016/S0140-6736(11)60890-8 22000136

[ref25] Doyon N. Prescott S. A. De Koninck Y. (2015). Mild Kcc2 hypofunction causes inconspicuous chloride dysregulation that degrades neural coding. Front. Cell. Neurosci. 9:516. doi: 10.3389/fncel.2015.00516 26858607 PMC4731508

[ref26] Dzhala V. I. Talos D. M. Sdrulla D. A. Brumback A. C. Mathews G. C. Benke T. A.. (2005). Nkcc1 transporter facilitates seizures in the developing brain. Nat. Med. 11, 1205–1213. doi: 10.1038/nm1301, PMID: 16227993

[ref27] Engel J. Jr. (2014). Approaches to refractory epilepsy. Ann. Indian Acad. Neurol. 17, S12–S17. doi: 10.4103/0972-2327.128644, PMID: 24791078 PMC4001229

[ref28] Esclapez M. Hirsch J. C. Khazipov R. Ben-Ari Y. Bernard C. (1997). Operative gabaergic inhibition in hippocampal Ca1 pyramidal neurons in experimental epilepsy. Proc. Natl. Acad. Sci. U. S. A. 94, 12151–12156. doi: 10.1073/pnas.94.22.12151, PMID: 9342378 PMC23733

[ref29] Fiest K. M. Sauro K. M. Wiebe S. Patten S. B. Kwon C. S. Dykeman J.. (2017). Prevalence and incidence of epilepsy: a systematic review and meta-analysis of international studies. Neurology 88, 296–303. doi: 10.1212/WNL.0000000000003509, PMID: 27986877 PMC5272794

[ref30] Frohlich F. Bazhenov M. Iragui-Madoz V. Sejnowski T. J. (2008). Potassium dynamics in the epileptic cortex: new insights on an old topic. Neuroscientist 14, 422–433. doi: 10.1177/1073858408317955, PMID: 18997121 PMC2854295

[ref31] Gagnon M. Bergeron M. J. Lavertu G. Castonguay A. Tripathy S. Bonin R. P.. (2013). Chloride extrusion enhancers as novel therapeutics for neurological diseases. Nat. Med. 19, 1524–1528. doi: 10.1038/nm.3356, PMID: 24097188 PMC4005788

[ref32] Goddard G. V. Mcintyre D. C. Leech C. K. (1969). A permanent change in brain function resulting from daily electrical stimulation. Exp. Neurol. 25, 295–330. doi: 10.1016/0014-4886(69)90128-9, PMID: 4981856

[ref33] Godoy L. D. Prizon T. Rossignoli M. T. Leite J. P. Liberato J. L. (2022). Parvalbumin role in epilepsy and psychiatric comorbidities: from mechanism to intervention. Front. Integr. Neurosci. 16:765324. doi: 10.3389/fnint.2022.765324 35250498 PMC8891758

[ref34] Gollwitzer S. Scott C. A. Farrell F. Bell G. S. De Tisi J. Walker M. C.. (2017). The long-term course of temporal lobe epilepsy: from unilateral to bilateral interictal epileptiform discharges in repeated video-EEG monitorings. Epilepsy Behav. 68, 17–21. doi: 10.1016/j.yebeh.2016.12.027 28109984

[ref35] Gutierrez R. Heinemann U. (2001). Kindling induces transient fast inhibition in the dentate gyrus--Ca3 projection. Eur. J. Neurosci. 13, 1371–1379. doi: 10.1046/j.0953-816x.2001.01508.x, PMID: 11298797

[ref36] Herrmann T. Gerth M. Dittmann R. Pensold D. Ungelenk M. Liebmann L.. (2021). Disruption of Kcc2 in parvalbumin-positive interneurons is associated with a decreased seizure threshold and a progressive loss of parvalbumin-positive interneurons. Front. Mol. Neurosci. 14:807090. doi: 10.3389/fnmol.2021.807090 35185464 PMC8850922

[ref37] Huusko N. Romer C. Ndode-Ekane X. E. Lukasiuk K. Pitkanen A. (2015). Loss of hippocampal interneurons and epileptogenesis: a comparison of two animal models of acquired epilepsy. Brain Struct. Funct. 220, 153–191. doi: 10.1007/s00429-013-0644-1, PMID: 24096381

[ref38] Kahle K. T. Khanna A. R. Duan J. Staley K. J. Delpire E. Poduri A. (2016). The Kcc2 cotransporter and human epilepsy: getting excited about inhibition. Neuroscientist 22, 555–562. doi: 10.1177/1073858416645087 27130838

[ref39] Kamphuis W. Gorter J. A. Da Silva F. L. (1991). A long-lasting decrease in the inhibitory effect of Gaba on glutamate responses of hippocampal pyramidal neurons induced by kindling epileptogenesis. Neuroscience 41, 425–431. doi: 10.1016/0306-4522(91)90338-O, PMID: 1870698

[ref40] Khazipov R. Valeeva G. Khalilov I. (2015). Depolarizing Gaba and developmental epilepsies. CNS Neurosci. Ther. 21, 83–91. doi: 10.1111/cns.12353, PMID: 25438879 PMC6495283

[ref41] Khirug S. Yamada J. Afzalov R. Voipio J. Khiroug L. Kaila K. (2008). Gabaergic depolarization of the axon initial segment in cortical principal neurons is caused by the Na-K-2Cl cotransporter Nkcc1. J. Neurosci. 28, 4635–4639. doi: 10.1523/JNEUROSCI.0908-08.2008, PMID: 18448640 PMC6670448

[ref42] Khurgel M. Switzer R. C. 3rd Teskey G. C. Spiller A. E. Racine R. J. Ivy G. O. (1995). Activation of astrocytes during epileptogenesis in the absence of neuronal degeneration. Neurobiol. Dis. 2, 23–35. doi: 10.1006/nbdi.1995.0003, PMID: 8980006

[ref43] Kim J. Shin H. K. Hwang K. J. Choi S. J. Joo E. Y. Hong S. B.. (2014). Mirror focus in a patient with intractable occipital lobe epilepsy. J. Epilepsy Res. 4, 34–37. doi: 10.14581/jer.14009, PMID: 24977131 PMC4066626

[ref44] Klorig D. C. Alberto G. E. Smith T. Godwin D. W. (2019). Optogenetically-induced population discharge threshold as a sensitive measure of network excitability. eNeuro:ENEURO.0229-18.2019:6. doi: 10.1523/ENEURO.0229-18.2019 PMC6838688 31619450

[ref45] Krook-Magnuson E. Armstrong C. Bui A. Lew S. Oijala M. Soltesz I. (2015). In vivo evaluation of the dentate gate theory in epilepsy. J. Physiol. 593, 2379–2388. doi: 10.1113/JP270056, PMID: 25752305 PMC4457198

[ref46] Kuruba R. Hattiangady B. Parihar V. K. Shuai B. Shetty A. K. (2011). Differential susceptibility of interneurons expressing neuropeptide Y or parvalbumin in the aged hippocampus to acute seizure activity. PLoS One 6:e24493. doi: 10.1371/journal.pone.0024493, PMID: 21915341 PMC3167860

[ref47] Lau D. Vega-Saenz De Miera E. C. Contreras D. Ozaita A. Harvey M. Chow A.. (2000). Impaired fast-spiking, suppressed cortical inhibition, and increased susceptibility to seizures in mice lacking Kv3.2 K+ channel proteins. J. Neurosci. 20, 9071–9085. doi: 10.1523/JNEUROSCI.20-24-09071.2000, PMID: 11124984 PMC6773003

[ref48] Liu Z. Luan G. Yang C. Guan Y. Liu C. Wang J.. (2020). Distinguishing dependent-stage secondary epileptogenesis in a complex case of giant hypothalamic hamartoma with assistance of a computational method. Front. Neurol. 11:478. doi: 10.3389/fneur.2020.00478, PMID: 32587568 PMC7297952

[ref49] Liu R. Wang J. Liang S. Zhang G. Yang X. (2019). Role of Nkcc1 and Kcc2 in epilepsy: from expression to function. Front. Neurol. 10:1407. doi: 10.3389/fneur.2019.01407 32010056 PMC6978738

[ref50] Löscher W. (2002). Animal models of epilepsy for the development of antiepileptogenic and disease-modifying drugs. A comparison of the pharmacology of kindling and post-status epilepticus models of temporal lobe epilepsy. Epilepsy Res. 50, 105–123. doi: 10.1016/S0920-1211(02)00073-6 12151122

[ref51] Löscher W. (2011). Critical review of current animal models of seizures and epilepsy used in the discovery and development of new antiepileptic drugs. Seizure 20, 359–368. doi: 10.1016/j.seizure.2011.01.003, PMID: 21292505

[ref52] Löscher W. Puskarjov M. Kaila K. (2013). Cation-chloride cotransporters Nkcc1 and Kcc2 as potential targets for novel antiepileptic and antiepileptogenic treatments. Neuropharmacology 69, 62–74. doi: 10.1016/j.neuropharm.2012.05.045, PMID: 22705273

[ref53] Lothman E. W. Williamson J. M. (1993). Rapid kindling with recurrent hippocampal seizures. Epilepsy Res. 14, 209–220. doi: 10.1016/0920-1211(93)90045-9, PMID: 8504791

[ref54] Luders H. O. (2001). Clinical evidence for secondary epileptogenesis. Int. Rev. Neurobiol. 45, 469–480. doi: 10.1016/S0074-7742(01)45024-0 11130912

[ref55] McNamara J. O. (1986). Kindling model of epilepsy. Adv. Neurol. 44, 303–318. 2871721

[ref56] Moore Y. E. Kelley M. R. Brandon N. J. Deeb T. Z. Moss S. J. (2017). Seizing control of Kcc2: a new therapeutic target for epilepsy. Trends Neurosci. 40, 555–571. doi: 10.1016/j.tins.2017.06.008, PMID: 28803659

[ref57] Morrell F. Tsuru N. Hoeppner T. J. Morgan D. Harrison W. H. (1975). Secondary epileptogenesis in frog forebrain: effect of inhibition of protein synthesis. Can. J. Neurol. Sci. 2, 407–416. doi: 10.1017/S0317167100020552, PMID: 1201528

[ref58] Niknazar M. Mousavi S. R. Motaghi S. Dehghani A. Vahdat B. V. Shamsollahi M. B.. (2013). A unified approach for detection of induced epileptic seizures in rats using EcoG signals. Epilepsy Behav. 27, 355–364. doi: 10.1016/j.yebeh.2013.01.028, PMID: 23542539

[ref59] Nogueira G. S. Santos L. E. Rodrigues A. M. Scorza C. A. Scorza F. A. Cavalheiro E. A.. (2015). Enhanced nonsynaptic epileptiform activity in the dentate gyrus after kainate-induced status epilepticus. Neuroscience 303, 59–72. doi: 10.1016/j.neuroscience.2015.06.057, PMID: 26141843

[ref60] Nusser Z. Hajos N. Somogyi P. Mody I. (1998). Increased number of synaptic Gaba(A) receptors underlies potentiation at hippocampal inhibitory synapses. Nature 395, 172–177. doi: 10.1038/25999, PMID: 9744275

[ref61] Okabe A. Yokokura M. Toyoda H. Shimizu-Okabe C. Ohno K. Sato K.. (2003). Changes in chloride homeostasis-regulating gene expressions in the rat hippocampus following amygdala kindling. Brain Res. 990, 221–226. doi: 10.1016/S0006-8993(03)03528-5 14568348

[ref62] Orman R. Von Gizycki H. Lytton W. W. Stewart M. (2008). Local axon collaterals of area Ca1 support spread of epileptiform discharges within Ca1, but propagation is unidirectional. Hippocampus 18, 1021–1033. doi: 10.1002/hipo.20460 18548581

[ref63] Otsu Y. Donneger F. Schwartz E. J. Poncer J. C. (2020). Cation-chloride cotransporters and the polarity of Gaba signaling in mouse hippocampal parvalbumin interneurons. J. Physiol. 598, 1865–1880. doi: 10.1113/JP279221 32012273

[ref64] Pallud J. Le Van Quyen M. Bielle F. Pellegrino C. Varlet P. Cresto N.. (2014). Cortical Gabaergic excitation contributes to epileptic activities around human glioma. Sci. Transl. Med. 6:244ra89. doi: 10.1126/scitranslmed.3008065 PMC4409113 25009229

[ref65] Papp E. Rivera C. Kaila K. Freund T. F. (2008). Relationship between neuronal vulnerability and potassium-chloride cotransporter 2 immunoreactivity in hippocampus following transient forebrain ischemia. Neuroscience 154, 677–689. doi: 10.1016/j.neuroscience.2008.03.072 18472345

[ref66] Pare D. Decurtis M. Llinas R. (1992). Role of the hippocampal-entorhinal loop in temporal lobe epilepsy: extra- and intracellular study in the isolated guinea pig brain in vitro. J. Neurosci. 12, 1867–1881. doi: 10.1523/JNEUROSCI.12-05-01867.1992, PMID: 1578275 PMC6575877

[ref67] Park K. M. Kim S. E. Lee B. I. Hur Y. J. (2018). Brain morphology in patients with genetic generalized epilepsy: its heterogeneity among subsyndromes. Eur. Neurol. 80, 236–244. doi: 10.1159/000496698 30661063

[ref68] Pathak H. R. Weissinger F. Terunuma M. Carlson G. C. Hsu F. C. Moss S. J.. (2007). Disrupted dentate granule cell chloride regulation enhances synaptic excitability during development of temporal lobe epilepsy. J. Neurosci. 27, 14012–14022. doi: 10.1523/JNEUROSCI.4390-07.2007 18094240 PMC2211568

[ref69] Pitkanen A. Lukasiuk K. Dudek F. E. Staley K. J. (2015). Epileptogenesis. Cold Spring Harb. Perspect. Med.:a022822:5. doi: 10.1101/cshperspect.a022822 26385090 PMC4588129

[ref70] Racine R. J. (1972). Modification of seizure activity by electrical stimulation. II. Motor seizure. Electroencephalogr. Clin. Neurophysiol. 32, 281–294. doi: 10.1016/0013-4694(72)90177-0, PMID: 4110397

[ref71] Raimondo J. V. Burman R. J. Katz A. A. Akerman C. J. (2015). Ion dynamics during seizures. Front. Cell. Neurosci. 9:419. doi: 10.3389/fncel.2015.00419 26539081 PMC4612498

[ref72] Righes Marafiga J. Vendramin Pasquetti M. Calcagnotto M. E. (2021). Gabaergic interneurons in epilepsy: more than a simple change in inhibition. Epilepsy Behav. 121:106935. doi: 10.1016/j.yebeh.2020.106935 32035792

[ref73] Rivera C. Li H. Thomas-Crusells J. Lahtinen H. Viitanen T. Nanobashvili A.. (2002). Bdnf-induced TrkB activation down-regulates the K+-Cl- cotransporter Kcc2 and impairs neuronal cl- extrusion. J. Cell Biol. 159, 747–752. doi: 10.1083/jcb.200209011, PMID: 12473684 PMC2173387

[ref74] Rivera C. Voipio J. Payne J. A. Ruusuvuori E. Lahtinen H. Lamsa K.. (1999). The K+/Cl- co-transporter Kcc2 renders Gaba hyperpolarizing during neuronal maturation. Nature 397, 251–255. doi: 10.1038/16697, PMID: 9930699

[ref76] Ryu B. Nagappan S. Santos-Valencia F. Lee P. Rodriguez E. Lackie M.. (2021). Chronic loss of inhibition in piriform cortex following brief, daily optogenetic stimulation. Cell Rep. 35:109001. doi: 10.1016/j.celrep.2021.109001, PMID: 33882304 PMC8102022

[ref77] Salanova V. Markand O. N. Worth R. (1994). Clinical characteristics and predictive factors in 98 patients with complex partial seizures treated with temporal resection. Arch. Neurol. 51, 1008–1013. doi: 10.1001/archneur.1994.00540220054014 7944998

[ref78] Sato M. (1976). A study of psychomotor epilepsy with "kindled" cat preparations. Folia Psychiatr. Neurol. Jpn. 30, 425–434. PMID: 136391 10.1111/j.1440-1819.1976.tb02279.x

[ref79] Schmidt D. Baumgartner C. Löscher W. (2004). Seizure recurrence after planned discontinuation of antiepileptic drugs in seizure-free patients after epilepsy surgery: a review of current clinical experience. Epilepsia 45, 179–186. doi: 10.1111/j.0013-9580.2004.37803.x, PMID: 14738426

[ref80] Sen A. Martinian L. Nikolic M. Walker M. C. Thom M. Sisodiya S. M. (2007). Increased Nkcc1 expression in refractory human epilepsy. Epilepsy Res. 74, 220–227. doi: 10.1016/j.eplepsyres.2007.01.004, PMID: 17344024

[ref81] Shapiro L. A. Wang L. Ribak C. E. (2008). Rapid astrocyte and microglial activation following pilocarpine-induced seizures in rats. Epilepsia 49, 33–41. doi: 10.1111/j.1528-1167.2008.01491.x, PMID: 18226170

[ref82] Shen Y. Gong Y. Ruan Y. Chen Z. Xu C. (2021). Secondary epileptogenesis: common to see, but possible to treat? Front. Neurol. 12:747372. doi: 10.3389/fneur.2021.747372, PMID: 34938259 PMC8686764

[ref83] Shimoda Y. Beppu K. Ikoma Y. Morizawa Y. M. Zuguchi S. Hino U.. (2022). Optogenetic stimulus-triggered acquisition of seizure resistance. Neurobiol. Dis. 163:105602. doi: 10.1016/j.nbd.2021.105602, PMID: 34954320

[ref84] Sloviter R. S. (1987). Decreased hippocampal inhibition and a selective loss of interneurons in experimental epilepsy. Science 235, 73–76. doi: 10.1126/science.2879352 2879352

[ref85] Sloviter R. S. (1994). The functional organization of the hippocampal dentate gyrus and its relevance to the pathogenesis of temporal lobe epilepsy. Ann. Neurol. 35, 640–654. doi: 10.1002/ana.410350604, PMID: 8210220

[ref86] Sloviter R. S. Sollas A. L. Barbaro N. M. Laxer K. D. (1991). Calcium-binding protein (calbindin-D28K) and parvalbumin immunocytochemistry in the normal and epileptic human hippocampus. J. Comp. Neurol. 308, 381–396. doi: 10.1002/cne.903080306, PMID: 1865007

[ref88] Tellez-Zenteno J. F. Hernandez-Ronquillo L. (2012). A review of the epidemiology of temporal lobe epilepsy. Epilepsy Res. Treat. 2012:630853. doi: 10.1155/2012/630853 22957234 PMC3420432

[ref89] Tsuru N. (1981). Phylogenesis and kindling. Folia Psychiatr. Neurol. Jpn. 35, 245–252. doi: 10.1111/j.1440-1819.1981.tb00222.x 7327465

[ref91] Van Erum J. Van Dam D. De Deyn P. P. (2019). Ptz-induced seizures in mice require a revised Racine scale. Epilepsy Behav. 95, 51–55. doi: 10.1016/j.yebeh.2019.02.029, PMID: 31026782

[ref92] Vargas J. R. Takahashi D. K. Thomson K. E. Wilcox K. S. (2013). The expression of kainate receptor subunits in hippocampal astrocytes after experimentally induced status epilepticus. J. Neuropathol. Exp. Neurol. 72, 919–932. doi: 10.1097/NEN.0b013e3182a4b266, PMID: 24042195 PMC3880830

[ref93] Viitanen T. Ruusuvuori E. Kaila K. Voipio J. (2010). The K+-Cl cotransporter Kcc2 promotes Gabaergic excitation in the mature rat hippocampus. J. Physiol. 588, 1527–1540. doi: 10.1113/jphysiol.2009.181826, PMID: 20211979 PMC2876807

[ref94] Virtanen M. A. Uvarov P. Hubner C. A. Kaila K. (2020). Nkcc1, an elusive molecular target in brain development: making sense of the existing data. Cells 9:2607. doi: 10.3390/cells9122607, PMID: 33291778 PMC7761970

[ref95] Wilder B. J. King R. L. Schmidt R. P. (1968). Comparative study of secondary epileptogenesis. Epilepsia 9, 275–289. doi: 10.1111/j.1528-1157.1968.tb04961.x, PMID: 4978018

[ref96] Wittner L. Erőss L. Czirják S. Halász P. Freund T. F. Maglóczky Z. S. (2005). Surviving Ca1 pyramidal cells receive intact perisomatic inhibitory input in the human epileptic hippocampus. Brain 128, 138–152. doi: 10.1093/brain/awh339 15548550

[ref97] Yoon H. H. Kwon H. L. Mattson R. H. Spencer D. D. Spencer S. S. (2003). Long-term seizure outcome in patients initially seizure-free after resective epilepsy surgery. Neurology 61, 445–450. doi: 10.1212/01.WNL.0000081226.51886.5B, PMID: 12939415

