# Supplementary figures and images for: Unilateral optogenetic kindling of hippocampus leads to more severe impairments of the inhibitory signaling in the contralateral hippocampus
Source: Front Mol Neurosci. 2023 Oct 24;16:1268311. doi: 10.3389/fnmol.2023.1268311 (PMC10627882; doi:10.3389/fnmol.2023.1268311)

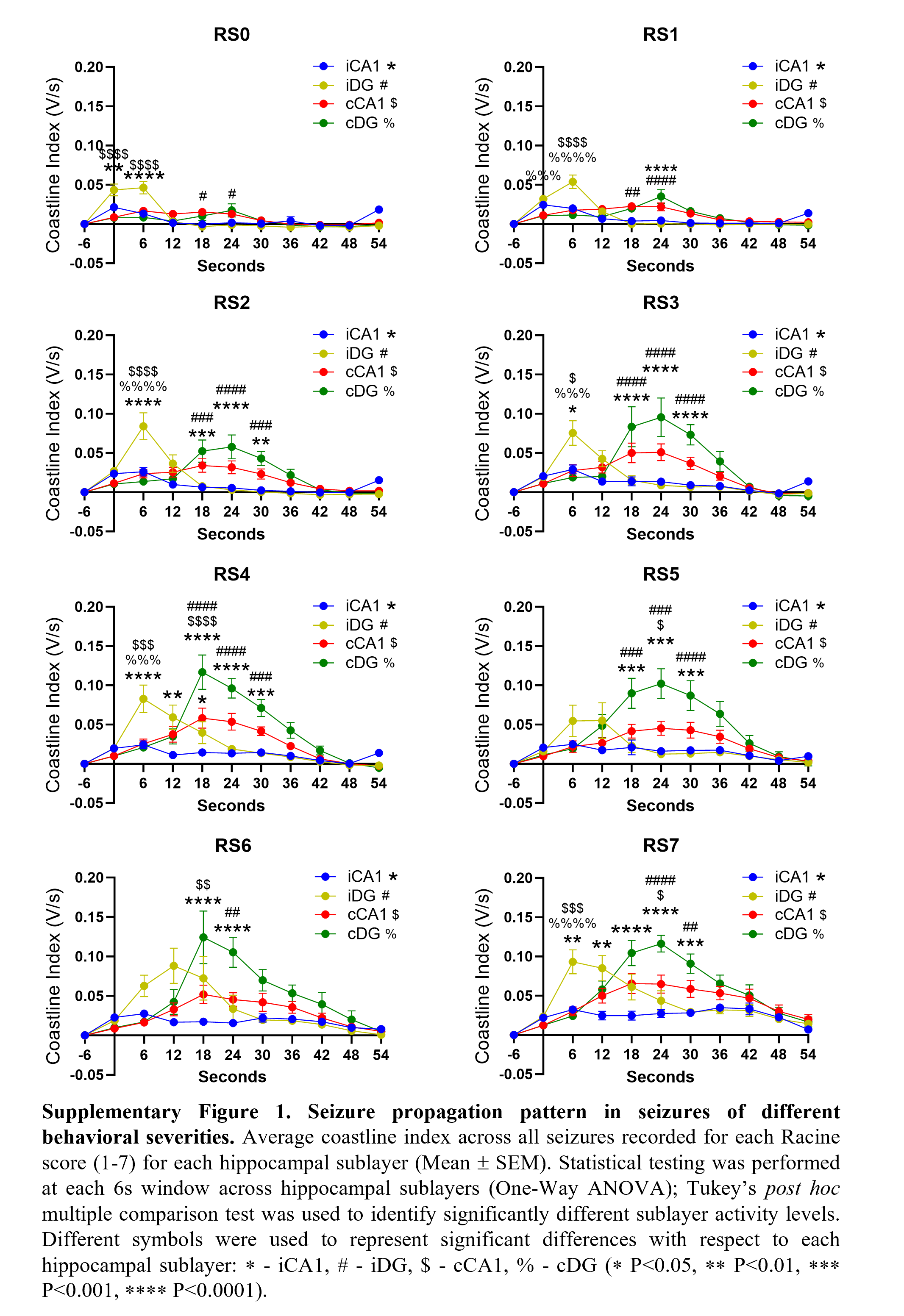

Supplement: Supplementary file 1 [file Image_1.tif]
